# Correction: Sustained Increase of 25-Hydroxyvitamin D Levels in Healthy Young Women during Wintertime after Three Suberythemal UV Irradiations—The MUVY Pilot Study

**DOI:** 10.1371/journal.pone.0169709

**Published:** 2017-01-03

**Authors:** The PLOS ONE Staff

## Notice of Republication

This article was republished on November 22, 2016, to correct errors in Fig 1 that were introduced during the technical check process. The publisher apologizes for the errors. Please download this article again to view the correct version. The originally published, uncorrected article and the republished, corrected articles are provided here for reference.

## Supporting Information

S1 File Originally published, uncorrected article. (PDF)

S2 File Republished, corrected article. (PDF)
