# Supplementary material for: Sustained Increase of 25-Hydroxyvitamin D Levels in Healthy Young Women during Wintertime after Three Suberythemal UV Irradiations—The MUVY Pilot Study
Source: PLoS One. 2016 Jul 19;11(7):e0159040. doi: 10.1371/journal.pone.0159040 (PMC4951026; doi:10.1371/journal.pone.0159040)
Supplement: S1 File — (PDF) [file pone.0159040.s003.pdf]

(11)

EA1/1/026

Antrag auf Beratung durch die Ethikkommission zur Durchführung eines medizinisch-wissenschaftlichen Vorhabens, welches nicht die klinische Prüfung eines Arzneimittels beinhaltet

1. Titel der Studie	**Vitamin-D_3-Synthese in der Haut bei UV-Bestrahlungen unter unterschiedlichen Bedingungen (Spektrum, Bestrahlungsstärke, Dosis, Hautfläche), unter Berücksichtigung von Strahlenschutzerfordernissen und bei Anwendung von Sonnenschutzmitteln**
2. Ethikkommissions -Antragsnummer	***EA1/026/09***
3. Entscheidungen anderer Ethikkommissionen in derselben Sache	Keine
4. Gegenstand der Studie und ihre Ziele; Angabe der Hypothesen, getrennt in Haupt- und Sekundärhypothesen sowie der klinischen Parameter (primäre und sekundäre Endpunkte), anhand derer die Hypothesen geprüft werden	Untersuchung der Vitamin-D3-Synthese durch UV-Hautbestrahlungen in Abhängigkeit von<br>(1) Expositionsbedingungen (Spektrum, Bestrahlungsstärke, Dosis, Anzahl, Größe der exponierten Fläche)<br>(2) individuellen Bedingungen (wie Alter, BMI)<br>(3) Strahlungsschwächungen durch Sonnenschutzmittel.
5. Erläuterung der Bedeutung der Studie	Die Ergebnisse sind Grundlage zur Erlangung hinreichender, für die Gesundheitsprävention empfohlener Vitamin-D-Konzentrationen durch UV-Hautbestrahlungen unter Berücksichtigung der Erfordernisse des Strahlenschutzes.
6. Welche der folgenden Bestimmungen finden Anwendung<br>a) Medizinproduktegesetz<br>-gemäß § 20 MPG (Gerät besitzt nicht die Konformitätserklärung oder diese liegt vor und es wird eine andere Indikation geprüft oder es werden zusätzlich invasive oder andere belastende Untersuchungen durchgeführt) **oder**<br>-gemäß § 23 MPG ?<br>b) Strahlenschutzverordnung § 23<br>c) Röntgenverordnung § 28 a<br>d) Gentechnikgesetz<br>e) Datenschutzgesetze	Zu a: Es werden ausschließlich kommerziell erhältliche, gemäß MPG für die UV-Heimtherapie zugelassene UV-Bestrahlungsgeräte und UV-Quellen eingesetzt.<br>Bestrahlungsgerät: Typ GH-8 ST (Waldmann GmbH)<br>UV-Quellen: Fluoreszenzröhren, Typ: ARIMED-B.<br><br>Zu b- d: nicht zutreffend.<br>Zu e: trifft zu.
7. Ggf.: Bezeichnung und Charakterisierung der Prüfprodukte (z.B. Geräte bei MPG-Studien; bitte Anlagen beifügen)	Kosmetische Hautpflege- und -schutzcreme in zwei Varianten mit je unterschiedlichen Sonnenschutzfaktoren (SPF 5 und SPF 15)<br>siehe Anlage 1
8. wesentliche Ergebnisse der vorklinischen Tests oder Gründe für die Nichtdurchführung derselben	(1) Verträglichkeitsnachweis und SPF-Bestimmung der eingesetzten Cremes durch unabhängiges Prüfinstitut im Auftrag des Herstellers (Beiersdorf AG), siehe Anlage 1<br>(2) Ärztlicher Eignungsnachweis für die Probanden, siehe Anlage 2: CRF.
9. Wesentlicher Inhalt und Ergebnisse der vorangegangenen Studien/Anwendungen der in der Studie zu prüfenden Produkte	(1) Dosis-Wirkungsuntersuchung zur Vitamin-D3-Synthese durch niedrig dosierte, sonnenähnliche UV-Strahlung bei Ganzkörperexposition ohne Sonnenschutzmittel, April 2008.
10. Beschreibung der vorgesehenen Maßnahmen/Untersuchungsmethoden und eventuelle Abweichungen von den in der med. Praxis üblichen Maßnahmen/Untersuchungen (was ist "Routine", was wird davon abweichend in der Studie gemacht?)	(1) Bestimmung des individuellen UV-Hauttyps<br>(2) Serielle UV-Exposition (sonnenähnliches Spektrum mit UVI=6 und UVI=8) von Gesicht, Dekolleté, Hände, Unterarmen mit ansteigender Dosis zwischen 25 % und 60 % der individuellen Erythemschwellendosis, insges.15 Bestrahlgen. Je Mo, Mi, Fr.

	(3) Vergleich von 4 Probandengruppen (G1:ohne Bestrahlung, G2: Bestr. Mit Creme 0, G3: Bestr mit Creme 1, G4: Bestr. Mit Creme 2), Creme 0: ohne SPF, Creme 2 mit SPF 4, Creme 3 mit SPF 15.<br>(4) Messungen:<br>- 7-DHC aus Hautschuppen (vor der Serie)<br>- Hautfarbe (nichtinvasiv, vor jeder Bestrahlung),<br>- 25(OH)D3, Kalzium, Phosphat aus Blutproben (1x pro Woche)<br>- Befindlichkeit (Fragebogen vor und nach jeder Bstrl.)
11. Bewertung und Abwägung der vorhersehbaren Risiken und Nachteile der Studienteilnahme gegenüber dem erwarteten Nutzen für die Studienteilnehmer und zukünftig erkrankte Personen (Nutzen-Risiko-Abwägung)	Vorhersehbare Risiken sind:<br>(1) Sonnenbrand bei UV-Überdosierung<br>(2) Photoallergische Reaktion<br>(3) Risiken der Blutabnahme<br>Die Risiken sind äußerst gering aufgrund<br>(1) Ausschluß gesundheitlich oder konstitutionell ungeeigneter Probanden<br>(2) UV-Bestrahlungen gemäß aktueller Schutz-empfehlungen der Strahlenschutzkommission mit erythemunterschwelligen Einzeldosen<br>(3) Vorprüfung der eingesetzten UV-Schutzcremes auf Unbedenklichkeit gegenüber Photoallergie<br>(4) Blutabnahme gemäß *state of the art*
29. Voraussehbarer therapeutischer Nutzen für die Studienteilnehmer (**individueller Nutzen** für den einzelnen Patienten)	Der einzelne Proband hat zunächst keinen Nutzen von der Studienteilnahme; dies muss erst durch die Studie gezeigt werden.<br>Aufwandsentschädigung: 300€ bzw. 100€ pro Teilnehmer bei abgeschlossene Studienteilnahme.
b. Voraussehbarer medizinischer Nutzen für zukünftig erkrankte Personen **(Gruppennutzen)**	Die Studie erfolgt an gesunden Erwachsenen. Der Nutzen der Ergebnisse liegt<br>(1) in der Prävention Vitamin-D-Mangel-bedingter Erkrankungen<br>(2) in der Prävention UV-bedingter Hauterkrankungen durch minimale einzeln und kumulativ dosierte UV-Hautbestrahlungen zur hinreichenden kutanen Vitamin-D3 Produktion bei Wahrung von UV-Hautschutzerfordernissen<br>(3) Anwendung optimierter Sonnenschutzmittel, die Vitamin-D3-Synthese ermöglichen, jedoch den Anforderungen zum UV-Hautschutz genügen.<br>(4) Risikosenkung der UV-/Heliotherapie durch Kenntnis minimal notwendiger Einzel- und Kumulativdosen, minimal erforderlicher Flächen zur hinreichenden der Dosis-Wirkungsbeziehungen
c. **Risiken** und Belastungen für die Studienteilnehmer (alle im Einzelnen auflisten)	(1) Risiken der UV-Hautbestrahlungen ergeben sich<br>- bei Nichteinhaltung der _usschlussbedingung<br>- bei Nichteinhaltung der Verhaltensanweisungen (Einnahme phototox./sesnibilisierender Subanzen, bei Erscheinen (abgeschminkt und<br>- bei Fehldosierungen infolge Nichteinhaltens der Anweisungen zum Eincremen<br>(2) Augenschädigung bei Bestrahlung ohne Schutzbrille<br>(3) Belastungen und Risiken der Blutabnahme

12. Maßnahmen zur Risikobeherrschung	- Probandenaufklärung<br>- Vorherige Testbestrahlung an kleiner Hautfläche ohne und mit Sonnenschutzcreme<br>- Kontrolle und Überwachung korrekten Eincremens, der Dosierung und der Bestrahlung und der Bestrahlungen durch Aufsichtspersonal<br>- Ausschluß von Probanden bei Nichteinhaltung der Verhaltensanweisungen<br>- Bestrahlung nur mit Schutzbrille<br>- Arzt im Hintergrund oder anwesend<br>- Bestrahlungen und Blutabnahmen nach *state of the art.*
13. Abbruchkriterien	- Zwischenzeitlicher Eintritt eines Ausschluß-kriteriums<br>- Nichteinhaltung der Verhaltensanweisungen<br>- photoallergische Reaktionen im exponierten Hautbereich<br>- Erkrankung<br>- Tod<br>- Ein unerwünschtes Ereignis, das als schwer genug erachtet wird, um ein Ausscheiden zu rechtfertigen<br>- Widerrufen der Einwilligung aus Gründen, die nicht mit einem unerwünschten Ereignis oder Unwirksamkeit zusammenhängen.
14. Anzahl, Alter und Geschlecht der betroffenen Personen	96 gesunde, freiwillige männliche und weibliche Probanden (+ 2 Probanden je Gruppe Überrekrutierung als Reserve)<br>4 Gruppen zu je 24 Probanden,<br>je 2 Untergruppen differenziert nach Alter, Hauttyp, BMI
15. Statistische Planung und Angabe sowie biometrische Begründung der Fallzahl und Unterschrift des/der Statistikers/Statistikerin<br>U. Pieh —	Es erfolgen Trendanalysen zur Untersuchungen der Dosis-Wirkungsbeziehungen der Vitamin-D3-Synthese sowie Signifikanztests zwischen den Untergruppen (UG); Probandenzahl je Gruppe: 24<br>UG 1: Alter 18 – 50 Jahre, UG 2: 60 – 80 Jahre je 12 Teilnehmer mit je 6 x Hauttyp I und II, 6 x Typ III, davon 3 x BMI < 25 und 3 x BMI ≥ 25<br><br>Verantwortliche Durchführung: Dr. H. Piazena
16.<br>a. Darlegung und ggf. Erläuterung der **Ein- und Ausschlusskriterien**	Siehe Anlage 3.
b. **Teilnehmerinformation** (wer diese mündlich erteilt und Angabe, wie viel Zeit zwischen Aufklärung und Einwilligung verbleibt, ansonsten Verweis auf deren Inhalt als Anlage möglich)	Siehe Anlage 4.
c. **Einwilligungserklärung** (Verweis auf deren Inhalt als Anlage möglich)	Siehe Anlage 5.
d. Ggf. **Information und Einwilligung des gesetzlichen Vertreters** (ggf. auch Beschreibung des Verfahrens zur Einrichtung einer gerichtlichen Betreuung)	Nicht zutreffend

17. Maßnahmen zur Gewinnung von Studienteilnehmern (Aushang ?, Zeitungsannoncen? Etc.)	- Annonce in der BerlinerZeitung, im Berliner Kurier und im Berliner Abendblatt<br>-freiwillige Meldung der Teilnehmer
18. Ggf.: **Grund für die Einbeziehung und Darlegung des therapeutischen Nutzens für Personen, die minderjährig und/oder nicht einwilligungsfähig sind.**	Nicht zutreffend, da ausschießlicher Einschluß von Erwachsenen
19. Beziehung zwischen Studienteilnehmer und Studienarzt/-ärztin (Ist der Studienarzt zugleich der behandelnde Arzt?)	Keine
20. Erklärung zur Einbeziehung möglicherweise vom Sponsor oder Studienarzt abhängiger Personen	Nicht zutreffend
21. Maßnahmen, die eine Feststellung zulassen, ob ein Studienteilnehmer an mehreren Studien zugleich oder vor Ablauf einer in der vorangegangenen Studie festgelegten Frist teilnimmt.	Persönliche Erklärung eines jeden Probanden zur Nichtteilnahme an gleichzeitig oder im Erfassungszeitraum laufenden anderen Studien.
22. Ggf.: Honorierung bzw. Kostenerstattung der Studienteilnehmer (Höhe, wofür soll gezahlt werden ?)	Honorar zur Teilnahme:<br>300 € je Teilnehmer der Gruppen mit Bestrahlung<br>100 € je Teilnehmer der Vergleichsgruppe ohne Bestrahlung
23. Ggf.: Plan für die Weiterbehandlung und medizinische Betreuung der betroffenen Personen nach dem Ende der Studie	Nicht vorgesehen
24. Ggf.: Versicherung der Studienteilnehmer (Versicherungsbestätigung und Versicherungsbedingungen, Versicherer, Versicherungsumfang, Versicherungsdauer)	Erfolgt über die Betriebshaftpflichtversicherung der Charité.
25. Ggf.: Dokumentationsverfahren (Verweis auf CRF-Bögen möglich)	Siehe CRF.
26. Ggf.: Beschreibung, wie der Gesundheitszustand gesunder betroffener Personen dokumentiert werden soll	(1) durch die ärztliche Eignungsuntersuchung<br>(2) durch Protokollführung im Verlauf der Bestrahlungen, Dokumentationen durch den Prüfarzt
27. Ggf.: Methoden, unerwünschte Ereignisse festzustellen, zu dokumentieren und mitzuteilen (wann, von wem und wie ??)	(1) Unerwünschte Ereignisse infolge der Untersuchungen werden durch den anwesenden Prüfarzt festgestellt und im Prüfprotokoll dokumentiert<br>(2) Ggf. werden Gegenmaßnahmen eingeleitet<br>(3) Unerwünschte Ereignisse außerhalb der Anwesenheitszeiten der Probanden in den Untersuchungsräumen mit möglicher Auswirkung auf die Ein-/_usschlussbedingungen müssen vom Probanden vor der Bestrahlung dem Prüfarzt zur Entscheidung/Regulierung mitgeteilt werden.
28. Vorgehen zum Schutz der Geheimhaltung der gespeicherten Daten, Dokumente und ggf. Proben, Darlegung der Verschlüsselung der Daten von Studienteilnehmern (*bitte nicht Initialen und*	- Pseudonymisierte Speicherung persönlicher wie erhobener Daten durch Zuordnung einer internen Nummer je Teilnehmer und einer externen (fortlaufenden) Nummer zur Weitergabe an Auftraggeber

Geburtsdatum als Codierungsschema verwenden!)	- Einsichtnahme von Vertreter des Sponsors in die personenbezogenen Daten nur im Beisein der Prüfärzte, es dürfen keine Kopien aus der Akte gezogen werden<br>- Vernichtung der Serumproben nach Ablauf Studie
29. Erklärung zur Einhaltung des Datenschutzes	**Die Datenschutzbestimmungen werden eingehalten.**
30. Namen und Anschriften der Einrichtungen, die als Studienzentrum oder Studienlabor in die Studie eingebunden sind, sowie der Studienleiter und die Studienärzte	Aus Platzgründen wurden zur Durchführung zwei Räume im City Point Center Lichtenberg, Weissenseer Weg 111, 10369 berlin als Außenstelle angemietet<br>Studienleiter: Dr. rer. nat. H. Piazena<br>Ärztlicher Leiter: Prof. Dr. med. R. Uebelhack<br>Prüfarzt: Prof. Dr. med. H. Meffert,<br>Frau Dr. Uebelhack
31. Angaben zur Eignung der Prüfstelle, insbesondere zur Angemessenheit der dort vorhandenen Mittel und Einrichtungen sowie des zur Durchführung der klinischen Prüfung zur Verfügung stehenden Personals und zu Erfahrungen in der Durchführung ähnlicher Studien	Die angemieteten Räume sind zur Durchführung angemessen. Die Infrastruktur (Bestrahlungsgeräte, Probandenliegen, Blutabnahmeset, Tische, Stühle, Garderoben) werden gestellt.<br>Das einbezogene Personal besteht aus ausgewiesenen Experten in den jeweiligen Bereichen.
32. Vereinbarung über den Zugang des Prüfers/Hauptprüfers/Leiter der klinischen Prüfung, zu den Daten und den Grundsätzen über die Publikation	**Die Ergebnisse werden publiziert.**
33. Angaben zur Finanzierung der Studie<br>(wir weisen auf § 263 StGB hin)	
a. Finanzierungsquelle (Name und Sitz)	(1) Prüfungen mit Sonnenschutzcreme: Beiersdorf AG, Hamburg<br>(2) Photobiologische Grundlagenuntersuchungen: interne Finanzierung.
b. Höhe der kalkulierten Kosten pro Teilnehmer und insgesamt	Gesamtkosten der Studie: 117.500 € / 104 Teilnehmer (einschl. Überrekrutierung), d.h.<br>1.130 € pro Teilnehmer
c. Höhe der Kostenerstattung pro Teilnehmer und insgesamt	300 € je Teilnehmer in den Bestrahlungsgruppen,<br>100 € je Teilnehmer in der unbestrahlten Vergleichsgruppe<br>27.800 € insgesamt.
