# Supplementary material for: Sustained Increase of 25-Hydroxyvitamin D Levels in Healthy Young Women during Wintertime after Three Suberythemal UV Irradiations—The MUVY Pilot Study
Source: PLoS One. 2016 Jul 19;11(7):e0159040. doi: 10.1371/journal.pone.0159040 (PMC4951026; doi:10.1371/journal.pone.0159040)
Supplement: S2 File — (DOCX) [file pone.0159040.s004.docx]

Trial study protocol approved by the ethics committee, translated from the original

Application for consultation from the Ethics committee for the realization of a medical-scientific project, which does not involve testing of pharmaceutical substances.

| 1. Study title | **Vitamin D3 synthesis in the skin under UV irradiation with different conditions (spectrum, radiation intensity, dosage, skin area), taking into account radiation protection requirements and application of sunscreens** |
| --- | --- |
| 2. Application number at the ethics committee | **EAl/026/09** |
| 3. Decision of other ethics committees for the same project | none |
| 4. Study content and objectives; Specification of hypotheses, separated into main and secondary hypothesis and the clinical parameters (primary and secondary outcome measures) used to test these hypotheses | Investigation of the Vitamin D3 synthesis by UV skin exposure in dependence of  (1) Exposure conditions (spectrum, intensity of irradiance, dosage, number of exposures, size of exposed area)  (2) Individual conditions (such as age and BMI)  (3) Attenuations of irradiation by sunscreen. |
| 5. Illustration of study relevance | The results are the basis for obtaining sufficient Vitamin D concentrations, recommended for health prevention by UV skin irradiation taking into account the requirements of the radiation protection. |
| 6. Which of the following provisions are applied?  a) Medicinal Devices Act § 20 or § 23  b) Radiation Protection Ordinance § 23  c) X-ray Ordinance § 28a  d) Act on Genetic Engineering  e) Data Privacy Act | a) Exclusively commercially available, UV irradiation devices and UV sources were used, according to MPG for UV home therapy approved.  Irradiation devices: Type GH-8 ST (Waldmann GmbH)  UV sources: fluorescent tubes, type: ARIMED-8.  b-d) Not applicable.  e) Is applied. |
| 7. If applicable: designation and characterization of the investigated product | Cosmetic skin care and protection cream in two versions, each with different sun protection factors (SPF 5 and SPF 15)  see attachment 1 |
| 8. Important results of preclinical tests and reasons for their non-conduction | (1) Compatibility verification and SPF determination of the applied creams by independent testing institute on behalf of the manufacturer (Beiersdorf AG), see attachment 1  (2) Physician’s proof of suitability of the participants, see attachment 2: CRF |
| 9. Important content and results of preceding trials/applications of the investigated products | (1) Dose-response investigation on Vitamin D3 synthesis by low-dose, sun-simulated UV irradiation with whole body exposure without sunscreen application, April of 2008. |
| 10. Description of preceding procedures/examination methods and possible deviations from the usually applied procedures/examinations in medical practice (what is the “routine”? What differs from that “routine” in this study?) | (1) Determination of the individual UV skin type  (2) Serial UV exposure (sun like spectrum with UVI = 6 and UVI = 8) of the face, cleavage, hands, forearms with increasing dosage between 25 % and 60% of individual erythema threshold dose, a total of 15 exposures. Every Mon, Wed, Fri  (3) Comparison of 4 groups of subjects (G1: without irradiation, G2: irradiation with cream 0, G3: irradiation with cream 1, G4: irradiation with cream 2), cream 0: without SPF, cream 1 with SPF 4, cream 2 with SPF 15 (4) Measurements: - 7-DHC of dander (before the series) - Skin color (non-invasive, before each irradiation) - 25 (OH) D3, calcium, phosphate from blood samples (1x per week) - mood (questionnaire before and after each irradiation) |
| 11. Assessment and evaluation of expected risks and disadvantages of taking part in the study compared to possible benefits for the participants or future patients (benefit-risk-analysis) | Foreseeable risks are:  (1) Sunburn from UV overdose  (2) Photo-allergic reaction  (3) Risks of venipuncture  The risks are extremely low due  (1) The exclusion of health wise or constitutionally unsuitable subjects  (2) UV irradiation with single sub-erythemal doses according to current protective recommendations of the Commission for Radiation Protection  (3) Preliminary examination of applied UV protection creams with regard to harmlessness for photo-allergic persons  4) State of the art venipunctures |
| a) Predictable therapeutical benefit for the participants (individual benefit for a single patient) | The individual subject has initially no benefit from participating in the study; a potential benefit is what needs to be detected first by conducting this study.  Expense allowance: 300 € or 100 € per participant after completing the study |
| b) Predictable medical benefit for future patients (group benefit) | The study is carried out in healthy adults. The benefit of the results lies  (1) In the prevention of Vitamin D deficiency-related diseases  (2) In the prevention of UV-induced skin diseases through UV skin irradiation in minimal singular and cumulative doses for adequate cutaneous Vitamin D3 production while ensuring the UV skin protection requirements  (3) Application of optimized sunscreens which allow Vitamin D3 synthesis but also meet the requirements for UV skin protection.  (4) Risk reduction of UV / Helio therapy through knowledge of minimal necessary single and cumulative doses, minimal required areas for sufficient dose-response relationships |
| c) Risks and burdens for the participants (specify in detail) | (1) Risks of UV skin exposures result from  - failure to comply with the exclusion condition  - Failure to comply with behavioral instructions (taking phototoxic / sensitizing substances, wearing make-up)  - At incorrect dosages due to noncompliance with the instructions on applying lotion  (2) Eye damage when irradiated without  safety glasses  (3) Burdens and risks of venipuncture |
| 12. Procedures for risk control | - Subscriber's control information  - Previous test exposure to a small skin area with and without sunscreen  - Controlling and monitoring of correct lotion application and irradiation dose, irradiations applied by supervisory staff  - Exclusion of subjects with noncomplying with the behavioral instructions  - Irradiation only with safety glasses  - Physician in the background or present  - State of the art irradiations and venipunctures |
| 13. Stop criteria | - Interim occurrence of an exclusion criterion  - Noncompliance with the behavioral instructions  - Photo-allergic reactions in the exposed skin area  - Disease  - Death  - An adverse event, which is considered severe enough to justify a resignation  - Repeal of consent for reasons not related to an adverse event or ineffectiveness |
| 14. Number, age and gender of the affected persons | 96 healthy, voluntary male and female subjects (+ 2 subjects per group for overrecruitment as a reserve): 4 groups of 24 subjects, each with 2 subgroups broken down by age, skin type, BMI |
| 15. Statistic planning, specification and biometric justification of number of cases and statiscian’s signature | Trend analyzes for investigations of dose-response relationships of the Vitamin D3 synthesis and significance tests between the subgroups (UG) are carried out; Number of participants per group: 24  UG 1: Age 18 - 50 years, UG 2: 60 - 80 years; 12 per participant per UG with 6 x skin type I and II, 6 x Type III, of which 3 x BMI <25 and 3 x BMI> 25  Responsible realization: Dr. H. Piazena |
| 16.  a) Display and if applicable explanation of criteria for in- and exclusion | See attachment 3 |
| b) Subscriber's control information (who gives them orally, declaration of time-span between information and agreement, also reference to enclosure possible) | See attachment 4 |
| c) Declaration of consent (reference to enclosure possible) | See attachment 5 |
| d) Information and consent of legal guardian | Not applicable |
| 17. Procedures for recruiting study participants (Noticeboard? Newspaper advertisement? Etc.) | -Advertisement in the following newspapers: Berliner Zeitung, Berliner Kurier, Berliner Abendblatt  -Voluntary commitment |
| 18. If applicable: reason for inclusion of underaged or incapacitated persons and demonstration of their therapeutic benefits | Not applicable |
| 19. Relationship between participants and study physician (Is the study physician also the attending physician?) | None |
| 20. Declaration of inclusion of persons potentially depending on sponsor or study physician | Not applicable |
| 21. Procedures to detect potential participation of study participants in several studies at the same time or participation before expiration of a determined deadline of a previous study | Personal declaration of each study participant not to participiate in any other studies at the same time |
| 22. If applicable: reward or reimbursement for study participants (amount, paid for what?) | Reward for participation:  300€ per participant in the irradiation group  100€ per participant in the control group without irradiation |
| 23. Plans for further treatment and medical care after the end of the study | Not intended |
| 24. If applicable: insurance of study participants  (insurance confirmation, insurance conditions, insurance provider, extent of insurance, insurance duration) | Trough business liability insurance of Charité Berlin |
| 25. If applicable: documentation procedure (reference to CRF sheet possible) | See CRF sheet |
| 26. Description of determination of health status in affected healthy persons | (1) With physician’s suitability examination  (2) With recording in writing during the irradiations, documentation by the study physician |
| 27. Methods to detect, document and report adverse events (when, from whom, how?) | (1) Adverse events resulting from the study are asserted and documented by the study physician  (2) If necessary remedial measure are initiated  (3) Adverse events outside the attendance times of the subjects in the examination facilities with possible impact on the in- or exclusion conditions must be reported to the study physician by the participants before exposure so that he/she can decide/regulate accordingly |
| 28. Procedures to protect the confidentiality of obtained data, documents and possible samples, Explanation of pseudonymization of study participants’ data (Please don’t use initials and birthdates as a code) | -Pseudonymized safing of personal as well as obtained data using an internal (consecutive) number and an external number to pass to sponsor  -Inspection of personal data by representatives of the sponsor only in presence of study physicians, copying files won’t be permitted  - Destruction of serum samples after study |
| 29. Declaration of compliance with data protection | **The data privacy policy is maintained** |
| 30. Names and addresses of facilities used as a study center or laboratory as well as the study director’s and the study physicians’ | For space reasons, two rooms at City Point Center Lichtenberg, Weissenseer Weg 111, 10369 Berlin, were rented as an outpost to conduct the study  Head of study: Dr. rer. nat. H. Piazena  Medical director: Prof. Dr. med R. Uebelhack.  Study physician: Prof. Dr. med H. Meffert.  Dr. Uebelhack |
| 31. Information on the suitability of the test center, in particular the appropriateness of its resources and facilities, and on the staff conducting this clinical trial and their experience in conducting similar studies | The rented rooms are appropriate for conducting the study. The necessary infrastructure (irradiation devices, stretchers, blood collection sets, tables, chairs, cloakrooms) is provided.  The staff involved consists of designated experts in their respective fields. |
| 32. Agreement on access of investigator/principal investigator/director of clinical trial to the data and publication policy | **Results will be published.** |
| 33. Declaration on study financing (we refer to § 263 SIGB) |  |
| a) Financing source (name and location) | (1) Trials including sunscreen: Beiersdorf AG, Hamburg  (2) Photobiological basic investigations: internally financed |
| b) Calculated expenses per participant and in total | Total cost of study: € 117500/ 104 participants (incl. overrecruitment), i.e. € 1,130 per participant |
| c) Reimbursement per participant and total amount of it | € 300 for each participant in the irradiation group, € 100 per participant in the unexposed control group  € 27,800 in total |
