# Supplementary material for: Sustained Increase of 25-Hydroxyvitamin D Levels in Healthy Young Women during Wintertime after Three Suberythemal UV Irradiations—The MUVY Pilot Study
Source: PLoS One. 2016 Jul 19;11(7):e0159040. doi: 10.1371/journal.pone.0159040 (PMC4951026; doi:10.1371/journal.pone.0159040)
Supplement: S3 File — (PDF) [file pone.0159040.s005.pdf]

RNT – IIT

MUVY-Pilotstudie

Ident-Nummer: I__I__I__I

Demographischer Teil

01. Datum: I__I__I.I__I__I.201__

02. Alter: I__I__I **03.** Geschlecht: ☐ weiblich ☐ männlich

04. Größe: I__I__I__I cm **05**. Gewicht: I__I__I,I__I kg

Menstruation:

06. Datum des 1. Tages der letzten Regelblutung: I__I__I.I__I__I.I__I__I

07. Einnahmen hormoneller Kontrazeptiva? ☐nein ☐ja

08. Wenn ja, welche? ____

Wenn nein: **09.** Zyklusdauer: ☐ regelmäßig ☐unregelmäßig

10. Länge eines Zyklus: ca. I__I__I Tage

Medikamente:

11. Regelmäßige Medikamenteneinnahmen? ☐ nein ☐ ja, welche

**12. Medikament**	**Tagesdosis**	**seit wann?**	**weshalb?**
____________________	____________	I__I__I. I__I__I.I__I__I__I__I	____________________
____________________	____________	I__I__I. I__I__I.I__I__I__I__I	____________________
____________________	____________	I__I__I. I__I__I.I__I__I__I__I	____________________
____________________	____________	I__I__I. I__I__I.I__I__I__I__I	____________________
____________________	____________	I__I__I. I__I__I.I__I__I__I__I	____________________
____________________	____________	I__I__I. I__I__I.I__I__I__I__I	____________________
____________________	____________	I__I__I. I__I__I.I__I__I__I__I	____________________

13. Einnahmen von Nahrungsergänzungsmitteln? ☐ nein ☐ ja, welche

**Ergänzungsmittel**	**Wie oft, Dosis?**	**seit wann?**	**weshalb?**
____________________	____________	I__I__I. I__I__I.I__I__I__I__I	____________________
____________________	____________	I__I__I. I__I__I.I__I__I__I__I	____________________
____________________	____________	I__I__I. I__I__I.I__I__I__I__I	____________________
____________________	____________	I__I__I. I__I__I.I__I__I__I__I	____________________
____________________	____________	I__I__I. I__I__I.I__I__I__I__I	____________________

14. Rauchen Sie? ☐ Nein ☐ Ja → ☐ gelegentlich ☐ täglich

Seit wie vielen Jahren? I__I__IJahr/e

Wie viele Zigaretten? I__I__I__I täglich

15. Haben Sie in der Vergangenheit geraucht? ☐ Nie

☐ Ja → Wie viele Jahre? |__|__|Jahr/e

Wie viele Zigaretten? |__|__|__| täglich

Erkrankungen:

16. Welche anderen Erkrankungen sind bei Ihnen bekannt (z.B. Bluthochdruck, Diabetes mellitus etc.)?

☐ keine

☐ folgende Erkrankungen:

____

____

17. Bestehen dermatologische Erkrankungen wie ansteckende Hauterkrankungen oder anormale Hautreaktionen auf UV-Licht?

☐ nein ☐ ja, welche________________________________

18. Ist bei Ihnen jemals ein Vitamin-D-Mangel diagnostiziert worden?

☐ ja ☐ nein

Wenn ja,

- ist es therapiert worden? ☐ ja ☐ nein
- womit erfolgte die Therapie?
- wie lange hat die Therapie gedauert?

19. Hatten Sie jemals eine Depression?

☐ ja ☐ nein

20. Benutzen Sie im Sommer beim Sonnenbad zwischen 10 und 15Uhr eine Sonnencreme mit Lichtschutzfaktor?

☐ immer

☐ oft

☐ gelegentlich

☐ selten

☐ nie

☐ ich nehme kein Sonnenbad im Sommer

- Creme mit welchem Lichtschutzfaktor benutzen Sie in der Regel?

21. Ist bei Ihren Angehörigen 1. (Eltern, Geschwister, Kinder) oder 2. Grades (Großeltern, Tante, Onkel, Cousin/e) jemals ein Vitamin-D-Mangel diagnostiziert worden?

☐ ja ☐ nein

- Wenn ja, bei wem?

22. Gibt/gab es bei Ihren Angehörigen 1. oder 2. Grades folgende Erkrankungen?

- Osteoporose ☐ ja ☐ nein
  - Wenn ja, bei wem?
- Rachitis ☐ ja ☐ nein
  - Wenn ja, bei wem?
- Osteomalazie ☐ ja ☐ nein
  - Wenn ja, bei wem?

23. Gibt/gab es bei Ihren Angehörigen 1. oder 2. Grades Krebserkrankung?

☐ ja ☐ nein

- wenn ja,

welche Krebserkrankung bei wem?

24. Gibt/gab es bei Ihren Angehörigen 1. oder 2. Grades Herz-Kreislauferkrankungen? (wie Bluthochdruck, Angina pectoris, Herzinfarkt, Schlaganfall)

☐ ja ☐ nein

- wenn ja,

welche Herz-Kreislauferkrankungen bei wem?

25. Gibt/gab es bei Ihren Angehörigen 1. oder 2. Grades Autoimmunerkrankungen? (wie Rheumatoide Arthritis, Diabetes mellitus Typ 1, chronische Darmerkrankungen)

☐ ja ☐ nein

- wenn ja,

welche Autoimmunerkrankungen bei wem?

Ernährung:

26. Wie oft essen Sie folgende Lebensmittel:

26.1 Eier

Ei, gekocht oder gebraten, Omelett (1 ganzes)	☐ Nie	☐1 mal pro Monat oder seltener	☐ 2-3 mal pro Monat	☐ 1-2 mal pro Woche	☐ 3-4 mal pro Woche	☐ 5 mal pro Woche oder häufiger
Eierpfannkuchen, Crêpes (2 Stück)	☐ Nie	☐ 1 mal pro Monat oder seltener	☐ 2-3 mal pro Monat	☐ 1-2 mal pro Woche	☐ 3-4 mal pro Woche	☐ 5 mal pro Woche oder häufiger

26.2 Fleisch und Wurstwaren

Leber (eine Portion)	☐ Nie	☐1 mal pro Monat oder seltener	☐ 2-3 mal pro Monat	☐ 1-2 mal pro Woche	☐ 3 mal pro Woche oder häufiger	
Leberwurst, Leberpastete (eine Portion)	☐ Nie	☐ 1 mal pro Monat oder seltener	☐ 2-3 mal pro Monat	☐ 1-2 mal pro Woche	☐ 3-4 mal pro Woche	☐ 5 mal pro Woche oder häufiger

26.3 Fisch

Fetter Fisch (Sardinen, Hering, Thunfisch, Matjeshering, Makrele, Lachs, Aal, Forelle) (eine Portion)	☐ Nie	☐1 mal pro Monat oder seltener	☐ 2-3 mal pro Monat	☐ 1-2 mal pro Woche	☐ 3 mal pro Woche oder häufiger
Fettarmer Fisch (Heilbutt, Seelachs, Zander, Scholle) (eine Portion)	☐ Nie	☐ 1 mal pro Monat oder seltener	☐ 2-3 mal pro Monat	☐ 1-2 mal pro Woche	☐ 3 mal pro Woche oder häufiger
Fischzubereitung (z. B. Fischfilet, -stäbchen, -frikadellen) (eine Portion)	☐ Nie	☐ 1 mal pro Monat oder seltener	☐ 2-3 mal pro Monat	☐ 1-2 mal pro Woche	☐ 3 mal pro Woche oder häufiger
Meeresfrüchte (z. B. Muscheln, Tintenfische, Garnelen, Krabben, Langusten, Hummer) (eine Portion)	☐ Nie	☐ 1 mal pro Monat oder seltener	☐ 2-3 mal pro Monat	☐ 1-2 mal pro Woche	☐ 3 mal pro Woche oder häufiger

26.4 Milch, Milchprodukte, Käse

Milch (150ml)	☐ Nie	☐1 mal pro Monat oder seltener	☐ 2-3 mal pro Monat	☐ 1-2 mal pro Woche	☐ 3-4 mal pro Woche	☐ 5-6 mal pro Woche	☐ 1-2 mal pro Tag	☐ 3 mal pro Tag oder häufiger

Kakao, Fruchtmilch (150ml)	☐ Nie	☐1 mal pro Monat oder seltener	☐ 2-3 mal pro Monat	☐ 1-2 mal pro Woche	☐ 3-4 mal pro Woche	☐ 5-6 mal pro Woche	☐ 1 mal pro Tag oder häufiger
Naturjoghurt, Buttermilch (150 g)	☐ Nie	☐1 mal pro Monat oder seltener	☐ 2-3 mal pro Monat	☐ 1-2 mal pro Woche	☐ 3-4 mal pro Woche	☐ 5-6 mal pro Woche	☐ 1 mal pro Tag oder häufiger
Dickmilch, Kefir (150 g)	☐ Nie	☐1 mal pro Monat oder seltener	☐ 2-3 mal pro Monat	☐ 1-2 mal pro Woche	☐ 3-4 mal pro Woche	☐ 5-6 mal pro Woche	☐ 1 mal pro Tag oder häufiger
Fruchtjoghurt (150 g)	☐ Nie	☐1 mal pro Monat oder seltener	☐ 2-3 mal pro Monat	☐ 1-2 mal pro Woche	☐ 3-4 mal pro Woche	☐ 5-6 mal pro Woche	☐ 1 mal pro Tag oder häufiger
Quark, Kräuterquark (kein Früchtequark) (1 Esslöffel, gehäuft)	☐ Nie	☐1 mal pro Monat oder seltener	☐ 2-3 mal pro Monat	☐ 1-2 mal pro Woche	☐ 3-4 mal pro Woche	☐ 5-6 mal pro Woche	☐ 1 mal pro Tag oder häufiger
Frischkäse (Rahmstufe, Doppelrahmstufe) (1 Esslöffel, gehäuft)	☐ Nie	☐1 mal pro Monat oder seltener	☐ 2-3 mal pro Monat	☐ 1-2 mal pro Woche	☐ 3-4 mal pro Woche	☐ 5-6 mal pro Woche	☐ 1 mal pro Tag oder häufiger
Molkenkäse (z. B. Hüttenkäse, Ricotta) (eine Portion)	☐ Nie	☐1 mal pro Monat oder seltener	☐ 2-3 mal pro Monat	☐ 1-2 mal pro Woche	☐ 3-4 mal pro Woche	☐ 5-6 mal pro Woche	☐ 1 mal pro Tag oder häufiger
Weichkäse (z. B. Camembert, Brie) (eine Portion)	☐ Nie	☐1 mal pro Monat oder seltener	☐ 2-3 mal pro Monat	☐ 1-2 mal pro Woche	☐ 3-4 mal pro Woche	☐ 5-6 mal pro Woche	☐ 1 mal pro Tag oder häufiger
Schnittkäse (z. B. Gouda, Emmentaler, Tilsiter, Edamer) (eine Portion)	☐ Nie	☐1 mal pro Monat oder seltener	☐ 2-3 mal pro Monat	☐ 1-2 mal pro Woche	☐ 3-4 mal pro Woche	☐ 5-6 mal pro Woche	☐ 1 mal pro Tag oder häufiger
Mozarella, Mascarpone, Feta (eine Portion)	☐ Nie	☐ 1 mal pro Monat oder seltener	☐ 2-3 mal pro Monat	☐ 1-2 mal pro Woche	☐ 3-4 mal pro Woche	☐ 5-6 mal pro Woche	☐ 1 mal pro Tag oder häufiger

26.5 Gemüse

Spinat, Mangold (eine Portion)	☐ Nie	☐1 mal pro Monat oder seltener	☐ 2-3 mal pro Monat	☐ 1-2 mal pro Woche	☐ 3 mal pro Woche oder häufiger
Sauerkraut (eine Portion)	☐ Nie	☐ 1 mal pro Monat oder seltener	☐ 2-3 mal pro Monat	☐ 1-2 mal pro Woche	☐ 3 mal pro Woche oder häufiger
Weiß-, Blumenkohl, Broccoli, Kohlrabi (eine Portion)	☐ Nie	☐ 1 mal pro Monat oder seltener	☐ 2-3 mal pro Monat	☐ 1-2 mal pro Woche	☐ 3 mal pro Woche oder häufiger

26.6 Fette/Öle

26.6.1 Wenn Sie Fleisch oder Fisch aßen, wie oft waren diese mit folgenden Fetten oder Ölen zubereitet?

Butter	☐ Nie	☐ Selten	☐ Häufig	☐ Immer	☐ Weiß nicht
Margarine	☐ Nie	☐ Selten	☐ Häufig	☐ Immer	☐ Weiß nicht
tierisches Kochfett (z. B. Schmalz, Speck)	☐ Nie	☐ Selten	☐ Häufig	☐ Immer	☐ Weiß nicht
Olivenöl	☐ Nie	☐ Selten	☐ Häufig	☐ Immer	☐ Weiß nicht
Sonnenblumen-, Keimöl oder anderes Öl	☐ Nie	☐ Selten	☐ Häufig	☐ Immer	☐ Weiß nicht

26.6.2 Wenn Sie Gemüse aßen, wie oft war es mit folgenden Fetten oder Ölen zubereitet bzw. wie oft wurden diese Fette oder Öle zugegeben ?

Butter	☐ Nie	☐ Selten	☐ Häufig	☐ Immer	☐ Weiß nicht
Margarine	☐ Nie	☐ Selten	☐ Häufig	☐ Immer	☐ Weiß nicht
tierisches Kochfett (z. B. Schmalz, Speck)	☐ Nie	☐ Selten	☐ Häufig	☐ Immer	☐ Weiß nicht
Olivenöl	☐ Nie	☐ Selten	☐ Häufig	☐ Immer	☐ Weiß nicht
Leinöl, Sonnenblumen-, Distel-, Keimöl oder anderes Öl	☐ Nie	☐ Selten	☐ Häufig	☐ Immer	☐ Weiß nicht

26.6.3 Brotaufstrich

Butter auf Brot	☐ Nie	☐ Selten	☐ Manchmal	☐ Häufig	☐ Immer
Margarine auf Brot	☐ Nie	☐ Selten	☐ Manchmal	☐ Häufig	☐ Immer
Halbfettmargarine auf Brot	☐ Nie	☐ Selten	☐ Manchmal	☐ Häufig	☐ Immer
Olivenöl auf Brot	☐ Nie	☐ Selten	☐ Manchmal	☐ Häufig	☐ Immer

27. Abschätzung des Hauttyps (nach RTS)

1	**Welchen Farbton weist Ihre unbestrahlte Haut auf?**		
	Rötlich	**1**	
	Weißlich	**2**	
	Leicht beige	**3**	
	Bräunlich	**4**	
2	**Hat Ihre Haut Sommersprossen?**		
	Ja, viele	**1**	
	Ja, einige	**2**	
	Ja, aber nur vereinzelt	**3**	
	Nein	**4**	
3	**Wie reagiert Ihre Gesichtshaut auf die Sonne ?**		
	Sehr empfindlich, meist Hautspannen	**1**	
	Empfindlich, teilweise Hautspannen	**2**	
	Normal empfindlich, nur selten Hautspannen	**3**	
	Unempfindlich, ohne Hautspannen	**4**	
4	**Wie lange können Sie sich im Frühsommer in Deutschland am Mittag bei wolkenlosem Himmel in der Sonne aufhalten, ohne einen Sonnenbrand zu bekommen?**		
	Weniger als 15 Minuten	**1**	
	Zwischen 15 und 25 Minuten	**2**	
	Zwischen 25 und 40 Minuten	**3**	
	Länger als 40 Minuten	**4**	
5	**Wie reagiert Ihre Haut auf ein längeres Sonnenbad?**		
	Stets mit einem Sonnenbrand	**1**	
	Meist mit einem Sonnenbrand	**2**	
	Oftmals mit einem Sonnenbrand	**3**	
	Selten oder nie mit Sonnenbrand	**4**	
6	**Wie wirkt sich bei Ihnen ein Sonnenbrand aus?**		
	Kräftige Rötung, teilweise schmerzhaft und Bläschenbildung, danach schält sich die Haut	**1**	
	Deutliche Rötung, danach schält sich die Haut	**2**	
	Rötung, danach schält sich die Haut manchmal	**3**	
	Fast nie Rötung und Hautschälen	**4**	

**7**	**Ist bei Ihnen nach einmaligem, längerem Sonnenbad anschließend ein Bräunungseffekt zu erkennen?**		
	Nie	**1**	
	Meist nicht	**2**	
	Oftmals	**3**	
	Meist	**4**	
**8**	**Wie entwickelt sich bei Ihnen die Hautbräunung nach wiederholtem Sonnenbad?**		
	Kaum oder gar keine Bräunung	**1**	
	Leichte Bräunung nach mehreren Sonnenbädern	**2**	
	Fortschreitende, deutlicher werdende Bräunung	**3**	
	Schnell einsetzende und tiefe Bräunung	**4**	
**9**	**Welche Angabe entspricht am ehesten Ihrer natürlichen Haarfarbe?**		
	Rot bis rötlich braun	**1**	
	Hellblond bis blond	**2**	
	Dunkelblond bis braun	**3**	
	Dunkelbraun bis schwarz	**4**	
**10**	**Welche Farbe haben Ihre Augen**		
	Hellblau, hellgrau oder hellgrün	**1**	
	Blau, grau oder grün	**2**	
	Hellbraun oder dunkelgrau	**3**	
	Dunkelbraun	**4**	
	**Summe (∑)**		
	**Geschätzter Hauttyp (= ∑/10)**		

Unterschrift: ………………………………………… Datum: ……………………………

28 Beck Depression Inventar (BDI) – Tag 1

Dieser Fragebogen enthält 21 Gruppen von Aussagen. Bitte lesen Sie jede Gruppe sorgfältig durch. Suchen Sie dann die Aussage in der Gruppe Heraus, die am Besten beschreibt, wie Sie sich in dieser Woche einschließlich heute gefühlt haben und kreuzen Sie die dazugehörige Ziffer (⓪①② oder ③) an. Falls mehrere Aussagen eine Gruppe zutreffen, können Sie auch mehrere Ziffern markieren. Lesen Sie auf jeden Fall alle Aussagen in jeder Gruppe, bevor Sie Ihre Wahl treffen.

A	☒
Ich bin nicht traurig.	⓪
Ich bin traurig.	①
Ich bin die ganze Zeit traurig und komme nicht davon los.	②
Ich bin so traurig, dass ich es kaum noch ertrage.	③

B	☒
Ich sehe nicht besonders mutlos in die Zukunft.	⓪
Ich sehe mutlos in die Zukunft.	①
Ich habe nichts, worauf ich mich freuen kann.	②
Ich habe das Gefühl dass die Zukunft hoffnungslos ist, und dass die Situation nicht besser werden kann.	③

C	☒
Ich fühle mich nicht als Versager.	⓪
Ich habe das Gefühl, öfter versagt zu haben, als der Durchschnitt.	①
Wenn ich auf mein Leben zurückblicke, sehe ich bloß eine Menge Fehlschläge.	②
Ich habe das Gefühl, as Mensch ein völliger Versager zu sein.	③

D	☒
Ich kann die Dinge genauso genießen, wie früher.	⓪
Ich kann die Dinge nicht mehr so genießen wie früher.	①
Ich kann aus nichts mehr eine echte Befriedigung ziehen.	②
Ich bin mit allem unzufrieden oder gelangweilt.	③

E	☒
Ich habe keine Schuldgefühle.	⓪
Ich habe häufig Schuldgefühle.	①
Ich habe fast immer Schuldgefühle.	②
Ich habe immer Schuldgefühle.	③

F	☒
Ich habe nicht das Gefühl, gestraft zu sein.	⓪
Ich habe das Gefühl, vielleicht bestraft zu werden.	①
Ich erwarte, bestraft zu werden.	②
Ich habe das Gefühl, bestraft zu sein.	③

G	☒
Ich bin nicht von mir enttäuscht.	⓪
Ich bin von mir enttäuscht.	①
Ich finde mich fürchterlich.	②
Ich hasse mich.	③

H	☒
Ich habe nicht das Gefühl, schlechter zu sein als alle anderen.	⓪
Ich kritisiere mich wegen meiner Fehler und Schwächen.	①
Ich mache mir die ganze Zeit Vorwürfe wegen meiner Mängel.	②
Ich gebe mir für alles die Schuld, was schief geht.	③

I	☒
Ich denke nicht daran, mir etwas anzutun.	⓪
Ich denke manchmal an Selbstmord, aber ich würde es nicht tun.	①
Ich möchte mich am liebsten umbringen.	②
Ich würde mich umbringen, wenn ich die Gelegenheit hätte.	③

J	☒
Ich weine nicht öfter als früher.	⓪
Ich weine jetzt mehr als früher.	①
Ich weine jetzt die ganze Zeit.	②
Früher konnte ich weinen, aber jetzt kann ich es nicht mehr, obwohl ich es möchte.	③

Punkte subtotal Seite 1: ____

K	☒
Ich bin nicht reizbarer als sonst.	⓪
Ich bin jetzt leichter verärgert oder gereizt als früher.	①
Ich fühle mich dauernd gereizt.	②
Die Dinge, die mich früher geärgert haben, berühren mich kaum mehr.	③

L	☒
Ich habe nicht das Interesse an Menschen verloren.	⓪
Ich interessiere mich jetzt weniger für Menschen als früher.	①
Ich habe mein Interesse an anderen Menschen verloren.	②
Ich habe mein ganzes Interesse an anderen Menschen verloren.	③

M	☒
Ich bin so entschlussfreudig wie immer.	⓪
Ich schieb Entscheidungen jetzt öfter als früher auf.	①
Es fällt mir schwerer als früher Entscheidungen zu treffen.	②
Ich kann überhaupt keine Entscheidungen mehr treffen.	③

N	☒
Ich habe nicht das Gefühl, schlechter auszusehen als früher.	⓪
Ich mache mir Sorgen, dass ich alt oder unattraktiv aussehe.	①
Ich habe das Gefühl, dass Veränderungen in meinem Aussehen auftreten, die mich hässlich machen.	②
Ich finde mich hässlich.	③

O	☒
Ich kann so gut arbeiten wie früher.	⓪
Ich muss mir einen Ruck geben, bevor ich eine Tätigkeit in Angriff nehme.	①
Ich muss mich zu jeder Tätigkeit zwingen.	②
Ich bin unfähig zu arbeiten.	③

P	☒
Ich schlafe so gut wie sonst.	⓪
Ich schlafe nicht mehr so gut wie früher.	①
Ich wache 1 bis 2 Stunden früher auf als sonst, und es fällt mir schwer wieder einzuschlafen.	②
Ich wache mehrere Stunden früher auf als sonst und kann nicht mehr einschlafen.	③

Q	☒
Ich ermüde nicht stärker als sonst.	⓪
Ich ermüde schneller als früher.	①
Fast alles ermüdet mich.	②
Ich bin zu müde, um etwas zu tun.	③

R	☒
Mein Appetit ist nicht schlechter als sonst.	⓪
Mein Appetit ist nicht mehr so gut wie früher.	①
Mein Appetit hat sehr stark nachgelassen.	②
Ich habe überhaupt keinen Appetit mehr.	③

S	☒
Ich habe in letzter Zeit kaum abgenommen.	⓪
Ich habe mehr als 2 Kilo abgenommen.	①
Ich habe mehr als 5 Kilo abgenommen.	②
Ich habe mehr als 8 Kilo abgenommen	③

Ich esse absichtlich weniger, um abzunehmen: JA NEIN

T	☒
Ich mache mir keine größeren Sorgen um meine Gesundheit als sonst.	⓪
Ich mache mir Sorgen um körperliche Probleme, wie Schmerzen, Magenbeschwerden oder Verstopfung.	①
Ich mache mir so große Sorgen über gesundheitliche Probleme, dass es mir schwer fällt, an etwas anderes zu denken.	②
Ich mache mir so große Sorgen über gesundheitliche Probleme, dass ich an nichts anderes mehr denken kann.	③

U	☒
Ich habe in letzter Zeit keine Veränderung meines Interesses an Sex bemerkt.	⓪
Ich interessiere mich weniger für Sex als früher.	①
Ich interessiere mich jetzt viel weniger für Sex.	②
Ich habe das Interesse an Sex völlig verloren.	③

Subtotal Punkte Seite 2: ________
Subtotal Punkteübertrag Seite 1: ________

Summenwerte: ________

29 Profile of Mood States (POMS) – Tag 1

Die folgenden Worte beschreiben verschiedene Gefühle. Bitte kreuzen Sie für jedes Wort den Kreis ⊗ an, der am besten Ihre Gefühle während der vergangenen 24 Stunden beschreibt.

Seit gestern fühle ich mich:

		0<br>= gar nicht	1<br>= etwas	2<br>=ziemlich	3<br>=stark	4<br>=sehr stark
1	zornig	○	○	○	○	○
2	abgeschlafft	○	○	○	○	○
3	unglücklich	○	○	○	○	○
4	lebhaft	○	○	○	○	○
5	unsicher	○	○	○	○	○
6	lustlos	○	○	○	○	○
7	traurig	○	○	○	○	○
8	aktiv	○	○	○	○	○
9	gereizt	○	○	○	○	○
10	verdrießlich	○	○	○	○	○
11	betrübt	○	○	○	○	○
12	energisch	○	○	○	○	○
13	ängstlich	○	○	○	○	○
14	hoffnungslos	○	○	○	○	○
15	überreizt	○	○	○	○	○
16	müde	○	○	○	○	○
17	verärgert	○	○	○	○	○
18	entmutigt	○	○	○	○	○
19	neidisch	○	○	○	○	○
20	fröhlich	○	○	○	○	○
21	verbittert	○	○	○	○	○
22	erschöpft	○	○	○	○	○
23	schwermütig	○	○	○	○	○
24	verzweifelt	○	○	○	○	○
25	träge	○	○	○	○	○
26	hilflos	○	○	○	○	○
27	ermattet	○	○	○	○	○
28	munter	○	○	○	○	○
29	wütend	○	○	○	○	○
30	schwungvoll	○	○	○	○	○
31	schlecht gelaunt	○	○	○	○	○
32	minderwertig	○	○	○	○	○
33	erschreckt	○	○	○	○	○
34	tatkräftig	○	○	○	○	○
35	entkräftet	○	○	○	○	○
36	nervös	○	○	○	○	○
37	ruhig	○	○	○	○	○
38	depressiv	○	○	○	○	○
39	besorgt	○	○	○	○	○
40	heiter	○	○	○	○	○
41	gut drauf	○	○	○	○	○
42	hungrig	○	○	○	○	○
43	gesprächig	○	○	○	○	○
44	glücklich	○	○	○	○	○
45	schläfrig	○	○	○	○	○
46	schmerzgeplagt	○	○	○	○	○

30 Beck Depression Inventar (BDI) – Tag 8

Dieser Fragebogen enthält 21 Gruppen von Aussagen. Bitte lesen Sie jede Gruppe sorgfältig durch. Suchen Sie dann die Aussage in der Gruppe Heraus, die am Besten beschreibt, wie Sie sich in dieser Woche einschließlich heute gefühlt haben und kreuzen Sie die dazugehörige Ziffer (⓪①② oder ③) an. Falls mehrere Aussagen eine Gruppe zutreffen, können Sie auch mehrere Ziffern markieren. Lesen Sie auf jeden Fall alle Aussagen in jeder Gruppe, bevor Sie Ihre Wahl treffen.

A	☒
Ich bin nicht traurig.	⓪
Ich bin traurig.	①
Ich bin die ganze Zeit traurig und komme nicht davon los.	②
Ich bin so traurig, dass ich es kaum noch ertrage.	③

B	☒
Ich sehe nicht besonders mutlos in die Zukunft.	⓪
Ich sehe mutlos in die Zukunft.	①
Ich habe nichts, worauf ich mich freuen kann.	②
Ich habe das Gefühl dass die Zukunft hoffnungslos ist, und dass die Situation nicht besser werden kann.	③

C	☒
Ich fühle mich nicht als Versager.	⓪
Ich habe das Gefühl, öfter versagt zu haben, als der Durchschnitt.	①
Wenn ich auf mein Leben zurückblicke, sehe ich bloß eine Menge Fehlschläge.	②
Ich habe das Gefühl, as Mensch ein völliger Versager zu sein.	③

D	☒
Ich kann die Dinge genauso genießen, wie früher.	⓪
Ich kann die Dinge nicht mehr so genießen wie früher.	①
Ich kann aus nichts mehr eine echte Befriedigung ziehen.	②
Ich bin mit allem unzufrieden oder gelangweilt.	③

E	☒
Ich habe keine Schuldgefühle.	⓪
Ich habe häufig Schuldgefühle.	①
Ich habe fast immer Schuldgefühle.	②
Ich habe immer Schuldgefühle.	③

F	☒
Ich habe nicht das Gefühl, gestraft zu sein.	⓪
Ich habe das Gefühl, vielleicht bestraft zu werden.	①
Ich erwarte, bestraft zu werden.	②
Ich habe das Gefühl, bestraft zu sein.	③

G	☒
Ich bin nicht von mir enttäuscht.	⓪
Ich bin von mir enttäuscht.	①
Ich finde mich fürchterlich.	②
Ich hasse mich.	③

H	☒
Ich habe nicht das Gefühl, schlechter zu sein als alle anderen.	⓪
Ich kritisiere mich wegen meiner Fehler und Schwächen.	①
Ich mache mir die ganze Zeit Vorwürfe wegen meiner Mängel.	②
Ich gebe mir für alles die Schuld, was schief geht.	③

I	☒
Ich denke nicht daran, mir etwas anzutun.	⓪
Ich denke manchmal an Selbstmord, aber ich würde es nicht tun.	①
Ich möchte mich am liebsten umbringen.	②
Ich würde mich umbringen, wenn ich die Gelegenheit hätte.	③

J	☒
Ich weine nicht öfter als früher.	⓪
Ich weine jetzt mehr als früher.	①
Ich weine jetzt die ganze Zeit.	②
Früher konnte ich weinen, aber jetzt kann ich es nicht mehr, obwohl ich es möchte.	③

Punkte subtotal Seite 1: ____

K	☒
Ich bin nicht reizbarer als sonst.	⓪
Ich bin jetzt leichter verärgert oder gereizt als früher.	①
Ich fühle mich dauernd gereizt.	②
Die Dinge, die mich früher geärgert haben, berühren mich kaum mehr.	③

L	☒
Ich habe nicht das Interesse an Menschen verloren.	⓪
Ich interessiere mich jetzt weniger für Menschen als früher.	①
Ich habe mein Interesse an anderen Menschen verloren.	②
Ich habe mein ganzes Interesse an anderen Menschen verloren.	③

M	☒
Ich bin so entschlussfreudig wie immer.	⓪
Ich schieb Entscheidungen jetzt öfter als früher auf.	①
Es fällt mir schwerer als früher Entscheidungen zu treffen.	②
Ich kann überhaupt keine Entscheidungen mehr treffen.	③

N	☒
Ich habe nicht das Gefühl, schlechter auszusehen als früher.	⓪
Ich mache mir Sorgen, dass ich alt oder unattraktiv aussehe.	①
Ich habe das Gefühl, dass Veränderungen in meinem Aussehen auftreten, die mich hässlich machen.	②
Ich finde mich hässlich.	③

O	☒
Ich kann so gut arbeiten wie früher.	⓪
Ich muss mir einen Ruck geben, bevor ich eine Tätigkeit in Angriff nehme.	①
Ich muss mich zu jeder Tätigkeit zwingen.	②
Ich bin unfähig zu arbeiten.	③

P	☒
Ich schlafe so gut wie sonst.	⓪
Ich schlafe nicht mehr so gut wie früher.	①
Ich wache 1 bis 2 Stunden früher auf als sonst, und es fällt mir schwer wieder einzuschlafen.	②
Ich wache mehrere Stunden früher auf als sonst und kann nicht mehr einschlafen.	③

Q	☒
Ich ermüde nicht stärker als sonst.	⓪
Ich ermüde schneller als früher.	①
Fast alles ermüdet mich.	②
Ich bin zu müde, um etwas zu tun.	③

R	☒
Mein Appetit ist nicht schlechter als sonst.	⓪
Mein Appetit ist nicht mehr so gut wie früher.	①
Mein Appetit hat sehr stark nachgelassen.	②
Ich habe überhaupt keinen Appetit mehr.	③

S	☒
Ich habe in letzter Zeit kaum abgenommen.	⓪
Ich habe mehr als 2 Kilo abgenommen.	①
Ich habe mehr als 5 Kilo abgenommen.	②
Ich habe mehr als 8 Kilo abgenommen	③

Ich esse absichtlich weniger, um abzunehmen: JA NEIN

T	☒
Ich mache mir keine größeren Sorgen um meine Gesundheit als sonst.	⓪
Ich mache mir Sorgen um körperliche Probleme, wie Schmerzen, Magenbeschwerden oder Verstopfung.	①
Ich mache mir so große Sorgen über gesundheitliche Probleme, dass es mir schwer fällt, an etwas anderes zu denken.	②
Ich mache mir so große Sorgen über gesundheitliche Probleme, dass ich an nichts anderes mehr denken kann.	③

U	☒
Ich habe in letzter Zeit keine Veränderung meines Interesses an Sex bemerkt.	⓪
Ich interessiere mich weniger für Sex als früher.	①
Ich interessiere mich jetzt viel weniger für Sex.	②
Ich habe das Interesse an Sex völlig verloren.	③

Subtotal Punkte Seite 2: ________
Subtotal Punkteübertrag Seite 1: ________

Summenwerte: ________

31 Profile of Mood States (POMS) – Tag 8

Die folgenden Worte beschreiben verschiedene Gefühle. Bitte kreuzen Sie für jedes Wort den Kreis ⊗ an, der am besten Ihre Gefühle während der vergangenen 24 Stunden beschreibt.

Seit gestern fühle ich mich:

		0 = gar nicht	1 = etwas	2 =ziemlich	3 =stark	4 =sehr stark
1	zornig	○	○	○	○	○
2	abgeschlafft	○	○	○	○	○
3	unglücklich	○	○	○	○	○
4	lebhaft	○	○	○	○	○
5	unsicher	○	○	○	○	○
6	lustlos	○	○	○	○	○
7	traurig	○	○	○	○	○
8	aktiv	○	○	○	○	○
9	gereizt	○	○	○	○	○
10	verdrießlich	○	○	○	○	○
11	betrübt	○	○	○	○	○
12	energisch	○	○	○	○	○
13	ängstlich	○	○	○	○	○
14	hoffnungslos	○	○	○	○	○
15	überreizt	○	○	○	○	○
16	müde	○	○	○	○	○
17	verärgert	○	○	○	○	○
18	entmutigt	○	○	○	○	○
19	neidisch	○	○	○	○	○
20	fröhlich	○	○	○	○	○
21	verbittert	○	○	○	○	○
22	erschöpft	○	○	○	○	○
23	schwermütig	○	○	○	○	○
24	verzweifelt	○	○	○	○	○
25	träge	○	○	○	○	○
26	hilflos	○	○	○	○	○
27	ermattet	○	○	○	○	○
28	munter	○	○	○	○	○
29	wütend	○	○	○	○	○
30	schwungvoll	○	○	○	○	○
31	schlecht gelaunt	○	○	○	○	○
32	minderwertig	○	○	○	○	○
33	erschreckt	○	○	○	○	○
34	tatkräftig	○	○	○	○	○
35	entkräftet	○	○	○	○	○
36	nervös	○	○	○	○	○
37	ruhig	○	○	○	○	○
38	depressiv	○	○	○	○	○
39	besorgt	○	○	○	○	○
40	heiter	○	○	○	○	○
41	gut drauf	○	○	○	○	○
42	hungrig	○	○	○	○	○
43	gesprächig	○	○	○	○	○
44	glücklich	○	○	○	○	○
45	schläfrig	○	○	○	○	○
46	schmerzgeplagt	○	○	○	○	○

32. Bestrahlungsprotokoll

UV-Ganzkörperbestrahlungen zur Stimulation der Vitamin D_3-Synthese

32.1 Hauttyp I

Bestrahlungen mit GH-8 ST (8 x Arimed-B (100W), <u>No.</u> ……….
Abstand = 39 cm, E_{er} = 0,37 W m^{-2}, 1 MED = 250 J m^{-2} (DIN 5050/1) , T_{er} = 11,26 min

<u>Ablauf:</u>

- **3 UV-Bestrahlungen pro Woche, jeweils Bauchseite und Rückenseite.**
- **1 Tag Pause zwischen zwei Bestrahlungen.**
- **Ausschlusskriterien aktuell überprüfen.**
- **keine Bestrahlung bei bestehendem UV-Erythem oder bereits erhaltener UV-Bestrahlung am gleichen Tag!**
- **Kosmetika einige Stunden vor der Bestrahlung entfernen!**
- **Schutzbrille aufsetzen!**
- **Dosis am Bestrahlungsgerät einstellen, Sollbestrahlungszeit wird angezeigt.**
- **Sollbestrahlungszeit ebenfalls am Kurzzeitwecker zur Kontrolle einstellen.**

Tag	Aktion	Datum	Erythemwirk-same Dosis		Expos.-dauer [min : s]	Bestrahlung Blutabnahme (BA)	
			[MED]	[J m^{-2}]		Datum/ Zeit	Aufsicht
1	1 Blut		-	-	-		
2	2 UV		0.32	80,0	3 : 36		
3	Pause		-	-	-		
4	3 UV		0.48	120,0	5 : 24		
5	Pause		-	-	-		
6	4 UV		0.60	150,0	6 : 45		
7	Pause		-	-	-		
8	Pause		-	-	-		
9	5 Blut		-	-	-		

32. Bestrahlungsprotokoll
UV-Ganzkörperbestrahlungen zur Stimulation der Vitamin D_3-Synthese

32.2 Hauttyp II/III

Bestrahlungen mit GH-8 ST (8 x Arimed-B (100W), No.: …………
Abstand = 39 cm, E_{er} = 0,37 W m^{-2}, 1 MED = 250 J m^{-2} (DIN 5050/1) , T_{er} = 11,26 min

Ablauf:

- **3 UV-Bestrahlungen pro Woche, jeweils Bauchseite und Rückenseite.**
- **1 Tag Pause zwischen zwei Bestrahlungen.**
- **Ausschlusskriterien aktuell überprüfen.**
- **keine Bestrahlung bei bestehendem UV-Erythem oder bereits erhaltener UV-Bestrahlung am gleichen Tag!**
- **Kosmetika einige Stunden vor der Bestrahlung entfernen!**
- **Schutzbrille aufsetzen!**
- **Dosis am Bestrahlungsgerät einstellen, Sollbestrahlungszeit wird angezeigt.**
- **Sollbestrahlungszeit ebenfalls am Kurzzeitwecker zur Kontrolle einstellen.**

Tag	Aktion	Soll-Datum	Erythemwirk-same Dosis		Expos.-dauer [min : s]	Bestrahlung Blutabnahme (BA)	
			[MED]	[J m^{-2}]		Datum/ Zeit	Aufsicht
1	1 Blut		-	-	-		
2	2 UV		0.40	100	4 : 30		
3	Pause		-	-	-		
4	3 UV		0.60	150,0	6 : 45		
5	Pause		-	-	-		
6	4 UV		0.75	187,5	8 : 27		
7	Pause		-	-	-		
8	Pause		-	-	-		
9	5 Blut		-	-	-		

33. Vitamin-D-Spiegel

**Tag**	**Aktion**	**Datum**	**25-Hydroxy-Vitamin D (in nmol/l)**	**1,25-Dihydroxy-Vitamin D (in pmol/l)**
**1**	Blutabnahme vor erster Bestrahlung			
**8**	Blutabnahme 3 Tage nach letzter Bestrahlung			
**36**	Blutabnahme nach 4 Wochen			
**50**	Blutabnahme nach 6 Wochen			

34. Unerwünschte Ereignisse/Nebenwirkungen

Sind bei Ihnen folgende unerwünschte Ereignisse oder Nebenwirkungen aufgetreten?

Nein ☐

Wenn ja, bitte markieren Sie welche und deren Schweregrad.

Unerwünschtes Ereignis	Grad 1	Grad 2	Grad 3	Grad 4	Grad 5
Schwindel	Leichte Unsicherheit oder Bewegungsempfinden	Mäßige Unsicherheit oder Bewegungsempfinden, Alltagstätigkeiten im sozialen und häuslichen Umfeld beschränkt	Schwere Unsicherheit oder Bewegungsempfinden, Alltagstätigkeiten zur Selbstversorgung beschränkt	-	-
Trockene Haut	Betrifft <10% der Körperoberfläche und kein Erythem oder Pruritus	Betrifft 10 - 30% Körperoberfläche und Erythem oder Pruritus vorhanden; Alltagstätigkeiten im sozialen und häuslichen Umfeld beschränkt	Betrifft >30% der Körperoberfläche und Pruritus vorhanden; Alltagstätigkeiten zur Selbstversorgung beschränkt	-	-
Erythem	-	Erythem (>90% Körperoberfläche) Keine assoziierten Symptome; Alltagstätigkeiten im sozialen und häuslichen Umfeld beschränkt	Erythem (>90% Körperoberfläche) mit assoziierten Symptomen (z.B. Pruritus oder Empfindlichkeit); Alltagstätigkeiten zur Selbstversorgung beschränkt	Erythem (>90% Körperoberfläche) mit assoziierter Flüssigkeit- oder Elektrolytabweichung; Intensivstation oder Zentrum für Schwerbrandverletzte	-
Hautschmerzen	Leichte Schmerzen	Mäßige Schmerzen; Alltagstätigkeiten im sozialen und häuslichen Umfeld beschränkt	Schwere Schmerzen; Alltagstätigkeiten zur Selbstversorgung beschränkt	-	-
Photosensibilität	schmerzloses Erythem (<10% Körperoberfläche)	Empfindliches Erythem (10 - 30% Körperoberfläche)	Erythem (>30% Körperoberfläche) mit Blasenbildung; Photosensibilität; orale Glucocorticoidtherapie indiziert; Schmerzlinderung indiziert (Schmerzmittel)	Lebensbedrohliche Konsequenzen; intensive Therapie indiziert	Tod
Pruritus	Leicht oder lokal begrenzt; lokale Therapie indiziert	Intensiv oder generalisiert; intermittierend; Kratzspuren; orale Therapie indiziert; Alltagstätigkeiten im sozialen und häuslichen Umfeld beschränkt	Intensiv oder generalisiert; dauernd vorhanden; Alltagstätigkeiten zur Selbstversorgung oder Schlaf beschränkt; orale Glucocorticoid- oder immunosuppressive Therapie indiziert	-	-
Hautatrophie	<10% Körperoberfläche; Assoziiert mit Teleangiektasien oder Veränderungen der Hautfarbe	10 - 30% Körperoberfläche; Assoziiert mit Dehnungsstreifen oder Verlust der Hautanhangsgebilde	>30% Körperoberfläche; Assoziiert mit Ulzera	-	-
Urtikaria	<10% Körperoberfläche; lokale Therapie indiziert	10 - 30% Körperoberfläche; orale Therapie indiziert	>30% Körperoberfläche; intravenöse Behandlung erforderlich	-	-

Übelkeit	Appetitlosigkeit, keine Änderung der Essgewohnheiten	Reduzierte Nahrungsaufnahme, kein signifikanter Gewichtsverlust, Dehydratation oder Mangelernährung	Unzureichende Nahrungs- oder Flüssigkeitsaufnahme; künstliche Ernährung indiziert	-	-

Sind bei Ihnen unerwünschte Ereignisse oder Nebenwirkungen aufgetreten, die Sie nicht in dieser Tabelle gefunden haben? Wenn ja, welche und wie stark ausgeprägt?

Unerwünschtes Ereignis	Grad 1 (gering/leicht)	Grad 2 (mäßig/deutlich)	Grad 3 (stark/ausgeprägt)	Grad 4 (lebensbedrohlich)

Wann sind die unerwünschten Ereignisse aufgetreten und wann sind die verschwunden? Haben Sie irgendwelche Maßnahmen (z.B. Salbe bei Hautrötung) gegen die unerwünschten Ereignisse unternommen?

Unerwünschtes Ereignis	Wann aufgetreten (Datum)	Wann verschwunden (Datum)	Maßnahmen
