# Supplementary material for: Correction: Sustained Increase of 25-Hydroxyvitamin D Levels in Healthy Young Women during Wintertime after Three Suberythemal UV Irradiations—The MUVY Pilot Study
Source: PLoS One. 2017 Jan 3;12(1):e0169709. doi: 10.1371/journal.pone.0169709 (PMC5207646; doi:10.1371/journal.pone.0169709)
Supplement: S2 File — (PDF) [file pone.0169709.s002.pdf]

PLOS | ONE

RESEARCH ARTICLE

Sustained Increase of 25-Hydroxyvitamin D Levels in Healthy Young Women during Wintertime after Three Suberythemal UV Irradiations—The MUVY Pilot Study

Maria Gudrun Biersack[1]*, Malgorzata Hajdukiewicz[1], Ralf Uebelhack[2], Leonora Franke[2], Helmut Piazena[3], Pascal Klaus[1], Vera Höhne-Zimmer[1], Tanja Braun[1], Frank Buttgereit[1], Gerd-Rüdiger Burmester[1], Jacqueline Detert[1]

1 Department of Rheumatology and Clinical Immunology, Charité–Universitätsmedizin Berlin, Germany, **2** Department of Psychiatry and Psychotherapy, Charité–Universitätsmedizin Berlin, Germany, **3** Medical Photobiology Group, Department of Internal Medicine, Charité–Universitätsmedizin Berlin, Germany

* maria.biersack@charite.de

CrossMark
click for updates

OPEN ACCESS

Citation: Biersack MG, Hajdukiewicz M, Uebelhack R, Franke L, Piazena H, Klaus P, et al. (2016) Sustained Increase of 25-Hydroxyvitamin D Levels in Healthy Young Women during Wintertime after Three Suberythemal UV Irradiations—The MUVY Pilot Study. PLoS ONE 11(7): e0159040. doi:10.1371/journal.pone.0159040

Editor: Judy R Rees, Geisel School of Medicine at Dartmouth, UNITED STATES

Received: November 2, 2015

Accepted: June 27, 2016

Published: July 19, 2016

Copyright: © 2016 Biersack et al. This is an open access article distributed under the terms of the Creative Commons Attribution License, which permits unrestricted use, distribution, and reproduction in any medium, provided the original author and source are credited.

Data Availability Statement: All relevant data are within the paper and its Supporting Information files.

Funding: The authors have no support or funding to report.

Competing Interests: The authors have declared that no competing interests exist.

Abstract

Objectives

Vitamin D (VitD) deficiency is a health problem prevalent not only in the elderly but also in young adults. The primary objective of our observational pilot study "MUVY" (**M**ood, **U**VR, **V**itamin D in **Y**oung women) was to test both the short-term and long-term effects of a series of three suberythemal UV radiation (UVR) exposures on the VitD status and well-being of young healthy women during winter in a repeat measure design.

Methods

20 healthy young women (Fitzpatrick skin types I–III, aged 21–25 years) received three full body broad band UVR exposures with an escalating erythemally weighted dose schedule during one week in winter, and completed self-report questionnaires monitoring symptoms of depression (Beck Depression Inventory, BDI) and affective state/well-being (Profile of Mood States, POMS) at baseline and three days after the last UVR exposure. 25-hydroxy-vitamin D (25(OH)D) and 1,25-dihydroxyvitamin D (1,25$(OH)_2$D) were measured in serum at baseline, and at study days 8, 36 and 50.

Results

Mean baseline 25(OH)D level was 54.3 nmol/L (standard deviation (s.d.) = 24.1), with seven women having VitD deficient status. Relevant symptoms of depression, as indicated by low BDI total scores (0–8), were absent. After the three UVR exposures the increment of 25(OH)D was an average of 13.9 nmol/L (95% confidence interval (CI) = 9.4–18.4) and 26.2 pmol/L (95%CI = 7.2–45.1) for 1,25$(OH)_2$D. Δ25(OH)D, and corresponding baseline levels were significantly and inversely associated (rho = -0.493, p = 0.027). Only 25(OH)D remained significantly increased above baseline for at least six weeks after the last UVR

exposure. A strong inverse correlation of the POMS subscale "Vigor/Activity" and the increment in 1,25$(OH)_2$D was found (rho = -0.739, p<0.001) at day 8.

Conclusions

Three suberythemal whole body UVR exposures during one week are a simple and suitable method for improving 25(OH)D levels during winter, for at least six weeks, and especially in young women with VitD deficient status.

Trial Registration

German Clinical Trials Register (Deutsches Register Kinischer Studien) DRKS00009274

Introduction

Vitamin D (VitD) is an important prohormone with numerous skeletal and non-skeletal functions [1]. Both forms of VitD, cholecalciferol ($VitD_3$) or ergocalciferol ($VitD_2$) are biologically inert and require an enzymatic conversion in the liver and kidney to produce its biological active hormonal form 1α,25-dihydroxyvitamin D (1,25$(OH)_2$D). Of note is that more than 35 additional VitD metabolites are formed by the body with possible biological functions [2–4].

Worldwide studies have shown that VitD deficiency occurs frequently across a wide range of populations and age groups [5] (compare [6] for German population). VitD deficiency is thought to be associated with autoimmune diseases, and the increased risk of developing hypertension and depression [7–11]. The causality of this relationship is not clear [12]. A number of mechanisms underlying the link between VitD deficiency and depressive disorders in adults have been proposed [13–16]. To date, the efficacy of VitD supplementation for preventing onset of depressive disorders or for reducing depressive symptoms remains insufficiently studied [12, 17–20].

Solar ultraviolet-B radiation reaching the Earth's surface (290–320 nm) is known to initiate VitD production in the skin, but also holds the risk of causing skin damage and carcinogenesis. Therefore, the recommendation for limiting summer sunlight exposure in order to prevent skin cancer may conflict with the need to have optimal VitD levels. On the other hand, because the cutaneous synthesis of VitD can provide 90% of the physiological supply [21], it is of interest that a number of experimental studies have provided data on the significance of dose, spectrum of UVR, area of irradiated body surface and skin pigmentation for VitD production and 25(OH)D levels in plasma [22–39]. Notably, a six-week study by Bogh et al. (2012) showed that full body narrow band UV-B exposure three times per week is more effective in treating VitD deficiency than a daily oral intake of 40 μg $VitD_3$ [40].

Based on these observations, our pilot study performed on healthy young women during the winter months aimed to investigate the short-term and long-term effects of just three suberythemal UVR exposures on the serum levels of 25(OH)D, and its biologically active metabolite 1,25$(OH)_2$D. 25(OH)D is the predominant circulating form of VitD, and is generally considered to be the best marker of VitD status [41].

25(OH)D has far less biological activity than 1,25$(OH)_2$D, which is formed mainly in the kidney, but also in other tissues and organs. According to Lips (2007), 1,25$(OH)_2$D is not suitable as a mean of assessing VitD status, because it keeps within reference limits as long as possible by a hormonal regulation [42]. Thus far, circulating 1,25$(OH)_2$D levels have received

relatively little attention, and previous studies of UV-induced VitD production have rarely included plasma 1,25$(OH)_2D$ in their assessments. Nevertheless, in subjects with VitD deficiency, the formation of 1,25$(OH)_2D$ may be limited because of reduced substrate availability for the 1α-hydroxylase (CYP27B1) in the kidney [43].

Previous studies of UV-B induced VitD production in the skin of relatively healthy subjects have not addressed the question as to whether UVR exposure during the winter months may also influence mood and well-being. Therefore, the secondary aim of our MUVY pilot study was to measure these possible effects by using standard self-rating questionnaires before and after the three UVR exposures. We hypothesized that just three suberythemal UVR exposures within one week would improve VitD status not only acutely but also for a longer time, and also, that the changes observed could be associated with positive effects on affective state and functioning.

Our pilot study was conducted in order to test the feasibility of procedures and assessments for later use in a subsequent larger research project. It should also generate important findings regarding the safety of the UVR dose selected for whole body irradiation with our device, response effects and variance of these effects as well the suitability of the self-report questionnaires selected.

At the time the MUVY pilot study was designed and performed, the results of another study were published, finding that a significant increase in 25(OH)D can be achieved with a very low UV-B dose [30].

Methods

The MUVY pilot study was part of a larger research project, entitled "Vitamin D3 synthesis in the skin under different conditions of UV radiation (spectrum, radiation intensity, dosage, skin area) taking into account radiation protection requirements and application of sunscreens" (compare S1 and S2 Files) which was approved by the Ethical Committee of the Charité-Universitätsmedizin Berlin on the 17th of February 2009 (EA1/026/09). The study protocol conforms to the ethical guides of the 1975 Declaration of Helsinki. Participants of the pilot study were recruited via advertisements posted at Berlin universities. Participants were informed about the goals and procedures of the pilot study, and gave written informed consent prior to enrolling. They were also paid for their participation.

The sample size required for a pilot study depends upon the balance between the level of precision that can be achieved and the costs incurred through data collection [44]. Adapted from the description by Johanson and Brooks [45], we calculated that a sample size no larger than between 10 and 20 would be appropriate for our pilot study with a repeated measure design, if the expected effect sizes for 25(OH)D increase (Cohen's d_z) would be between 1.0 and 0.7 with a correlation coefficient between repeated measures of 25(OH)D levels between 0.8 and 0.7.

This trial is registered at the German Registry for Clinical Trials (Deutsches Register Klinischer Studien): Registration Number (DRKS00009274) and URL (http://www.drks.de/DRKS00009274).

A TREND check list for nonrandomized trials is attached in supplemental file (S2 Chart, TREND check list).

The authors confirm that all related trials for this intervention will be registered.

Study participants

The study participants underwent evaluation and management at the Charité-Universitätsmedizin Berlin, Department of Rheumatology and Clinical Immunology. 20 healthy female

university students (aged 21–25 years) were included and examined in the winter months of December 2011 to March 2012, when daily solar UV-B radiation is minimal to negligible in Berlin, Germany (Latitude: 52° N) [46].

Subjects were screened for medical and psychological health via questionnaires and interviews, and were excluded if they had known dermatological, hepatic, renal or psychiatric disorders, namely depressive disorders, known inadequate reactions to sunlight (such as solar urticaria, polymorphic light eruption or photodermatitis), regularly took VitD supplementation or possibly photosensitizing medication, had recently visited sunnier countries or undergone solarium tanning, or were aged over 30 years. Only Caucasian women of Fitzpatrick skin types I, II and III living in Berlin were included. Of the initially interested 30 volunteers, 10 were not included due to either their taking of Vitamin D supplementation, having a history of inadequate reactions to sunlight and/or being Fitzpatrick skin type IV (compare Fig 1).

Procedure and Irradiation

The UV source was an approved phototherapeutic device for whole-body irradiation (Waldmann GmBH and Co, Germany, type GH-8 ST) fitted with eight broad-band UV fluorescent tubes, type ARIMED B® (100 W). The output of the tubes over the entire UV band from 250 to 400 nm is composed of 8% UVB (280–320 nm) and 92% UVA (320–400 nm) [47]. Within the spectral range of UV-B the spectral slope of emission of these tubes was approximately comparable to the spectral slope of solar irradiance at the Earth's surface during noon-time in summer in central Europe and under a cloudless sky.

Spectral irradiance emitted by the device was measured at the center of the area of skin exposure, and at a distance from the radiation output window which equaled the distance at which the skin of the volunteers was exposed. The measurements were performed in the spectral range 250–400 nm, with a spectral resolution of 1 nm and spectral steps of 1 nm, using a double-monochromator spectroradiometer (type: OL 754, Optronic Inc., Orlando, Fl., USA). This was equipped with an Ulbricht sphere as the optical head. Before starting the measurement, the spectroradiometer was calibrated using a mercury fluorescent lamp to correct the wavelength shift, and a 200 W tungsten halogen standard lamp traceable to NIST. Erythema effective irradiance at the target distance of 39 cm was calculated to be 0.37 W m^{-2} by weighting spectral irradiance data with the CIE erythema action spectrum and subsequent integration over the full spectral range measured [48]. This value was used to calculate the exposure times required to obtain UV doses as defined in our exposure schedule.

The dose schedule for UVR exposures in our study was in line with the recommendations of the UV radiation protection decree of the Federal Government of Germany [49]. All participants had three UV sessions with whole body exposure (90% of the body surface area) within one week at one day intervals. The radiation dose was expressed in units of the (erythemally weighted) Standard Erythema Dose (1 SED = 100 J/m^2).[48].

As the individual Minimal Erythema Doses (MED)s for broad band UVB of our study participants were not known, we started with low UV doses for safety reasons: 0.8 SED was the initial dose for women with skin type I (n = 2) and 1.0 SED for women with skin types II and III (n = 18). If the skin inspected 24 hours after exposure showed no erythema the dose was increased to 1.2 SED for skin type I, and 1.5 SED for skin types II and III on study day 3. On day 5 the participants were exposed to a UV dose of 1.5 SED for skin type I and 1.875 SED for skin types II and III.

Blood samples for VitD assays were obtained before the first UVR exposure (day 1) and three days after the last UVR exposure (day 8), then at days 36 and 50 (4.5 and 6.5 weeks after the last UVR exposure, respectively).

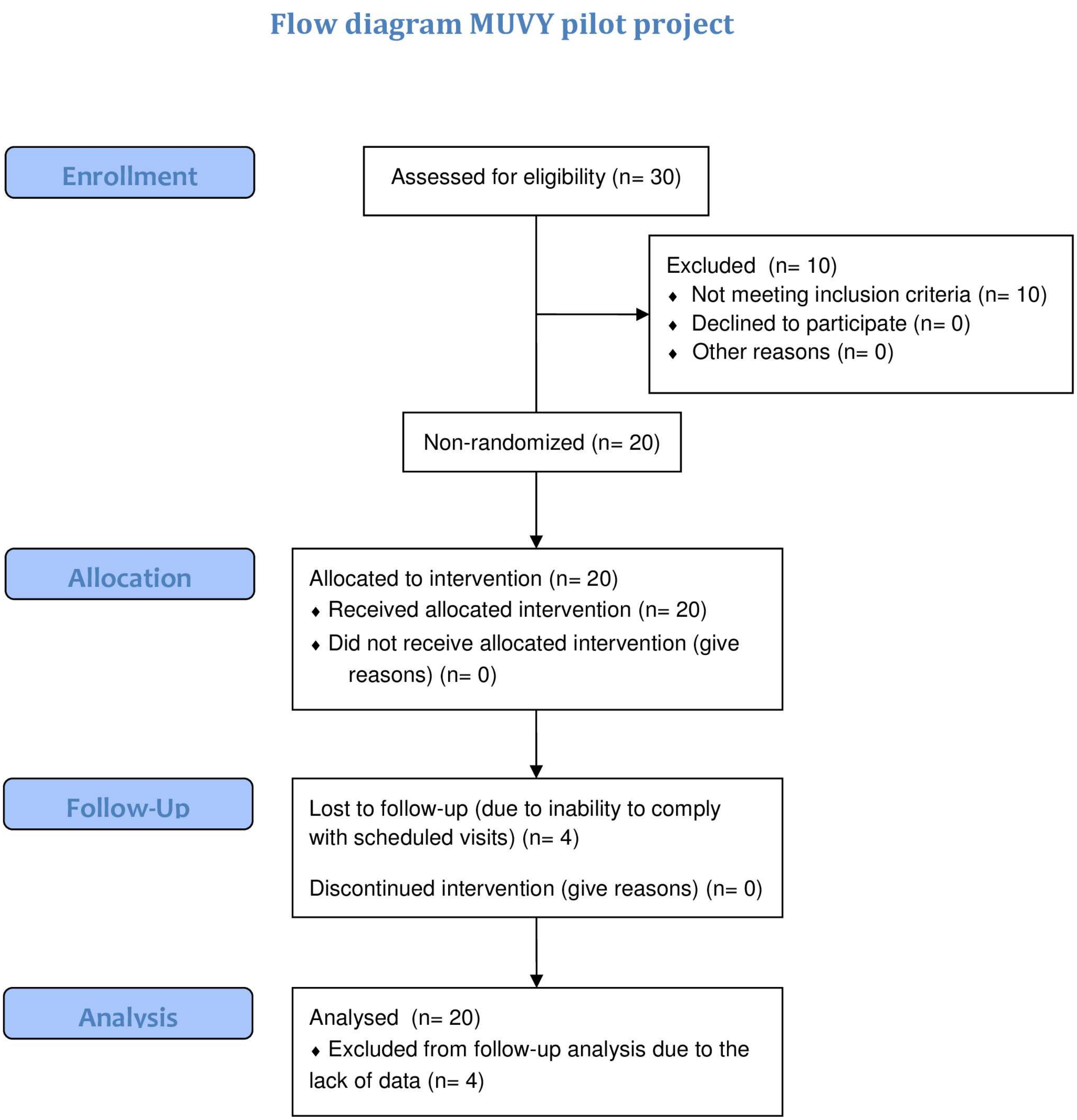

Fig 1. CONSORT flowchart.

doi:10.1371/journal.pone.0159040.g001

All 20 subjects completed the one week course of UVR exposures including day 8 assessments. 16 attended all follow-up examinations. Three women were absent for the day 36 follow-up blood sample, one of which was also absent for day 50. Another subject was absent as well on day 50. This was due to an inability to comply with scheduled visits (compare Fig 1).

The first date of enrollment was the 5th of December 2011. All data (including blood works results) was obtained by the 30th of May 2012.

Assessments

Estimation of skin type. To obtain a homogeneous group of women with respect to the skin photo type, we used the self-rating scale recommended by the UV radiation protection decree of the Federal Government of Germany [49]. This scale is similar in eight of the ten items to the Fitzpatrick skin type questionnaire: genetic disposition (color of eyes, hair, skin, existence of freckles) and reaction to sunlight (reaction of skin in general and facial skin to sunlight, degree of getting tanned) [50, 51]. The Fitzpatrick category „tanning habits"was replaced by questions on disposition for developing erythema [49].

Nutritional VitD intake status. All possible VitD containing food items in the "German Food Propensity Questionnaire" (GFPQ) of the German Institute of Human Nutrition Potsdam-Rehbrücke (DiFe, Deutsches Institut für Ernährungsforschung) were presented to the subjects, who were asked to specify the amount of these food items they consumed on average (for example "How many portions of fatty fish do you eat?", with the options: "never"to "three times per week and more") [52]. Portion sizes were defined using the AID (information service on nutrition, agriculture and consumer protection) chart of average portion sizes [53]. After converting frequency of consumed food portions into grams per day, the daily VitD intake was calculated using a nutritional content online calculator [54].

Assessment of affective state and well-being. To quantify affective state and levels of well-being, the study participants completed both questionnaires, at baseline, and three days after the last UVR exposure (study day 8):

The **Beck Depression Inventory (BDI)** was originally designed to measure the presence and intensity of typical symptoms of depression in patients who have already been diagnosed by a psychiatrist according to standard criteria. The BDI consists of 21 items that assess affective, cognitive and somatic symptoms of depression, rated on four-points scales.

The BDI is considered an adequate tool for ruling out clinically significant depressive disorders in different medical conditions [55, 56]. We applied the conventional cut-offs for classification of BDI scores into non-depressed (under 11) and depressed (11 or higher).

The Profile of Mood States (POMS), in its original version, is a 65-item inventory designed for use with adult psychiatric outpatients, which monitors affective state, levels of functioning, and subjective well-being [57]. The POMS assesses six dimensions of the mood construct: anger, confusion, depression, fatigue, tension, and vigor. In addition, "total mood disturbance" can be calculated by subtracting the Vigor subscale score from the sum of the tension, depression, anger, confusion, and fatigue subscale scores.

At present, there are varied short forms of the POMS in use. In the MUVY study, we used the well-validated German short form of the POMS [58]. It consists of 35 items measured on a 0-4 numerical rating scale with only four subscales (depression/anxiety, fatigue, hostility and vigor/activity). All participants were asked to rate "How are you feeling right now?" in terms of the 35 mood descriptions. According to the original version we focused on the calculation of the "total mood disturbance" score, by subtracting the vigor subscale score from the sum of the depression/anxiety, fatigue and hostility subscale scores. Thus, negative POMS total scores indicate the predominance of the vigor/activity subscale over the other subscales, whilst positive scores represent "mood disturbances" of different severity.

As indicated by low BDI scores at baseline, our study participants who received UVR were not classified as suffering from major or minor depression.

Blood collection and biochemical assessments. Venous blood samples (10 ml) were collected into Vacutainer® tubes without anticoagulants between 4 and 6 p.m., and stored for one hour at room temperature to ensure complete clotting. After centrifugation according to standard procedures, aliquots of serum were kept at -80°C until assayed. After the study was

finished, all serum samples were analyzed in one batch adhering to standard operating procedures at our hospital laboratory for clinical chemistry with external quality control (Labor Berlin–Charité Vivantes Service). The laboratory uses a new generation of automated, chemiluminescence-based immunoassays of 25(OH)D and 1,25(OH)2D (IDS-iSYS 25(OH)D and IDS-iSYS1,25(OH)2D, Immunodiagnostic Systems, Ltd, Boldon, UK).

The measurement range of these assays is 15–315 nmol/L for 25 (OH)D and 18–504 pmol/L for 1,25(OH)2D (information of the manufacturer). The IDS-iSYS control sets were used for quality control.

Although there is no universally accepted definition of VitD deficiency, insufficiency or optimal status, we classified our participants according to serum 25(OH)D concentrations categorized into three groups: <50 nmol/L (deficient); 50-75 nmol/L (insufficient), and >75 nmol/L (optimal) [41]. Generally, 25(OH)D levels >75 nmol/L are considered desirable for fracture prevention [59].

1,25$(OH)_2$D levels between 50.4 pmol/L and 245 pmol/L are given as the specific reference range by the laboratory conducting our analyses [60].

Adverse Events (AEs). Although suberythemal UVR exposures should not be associated with adverse skin effects, the participants were invited to complete AE forms during this trial. The following AEs such as dizziness, dry skin, erythroderma, pain of skin, occurrence of erythema (different grades), pruritus, skin atrophy, urticaria, and nausea were classified according to the "Common Terminology Criteria for Adverse Events" (CTCAE) version 4.0, and intensity, duration, and applied measures for improvement were noted [61]. Assessment of additional, unlisted AEs was possible and encouraged.

Statistical analyses

Data was analyzed using the statistical software package SPSS 18.0 (SPSS Institute, Chicago, USA). Descriptive statistics were inspected for normal distribution of variables using the Kolmogorov-Smirnov test, homogeneity of the variance (Levene-test) and outliers. The results are presented as mean±standard deviation (s.d.) or median.

Continuous variables were compared using the paired Student's t-test or Wilcoxon test for repeated measures. Whilst also controlling for putative confounders such as the use of oral contraceptives, skin type and daily dietary VitD intake, we also employed univariate general linear models (GLM) for the baseline 25(OH)D and 1,25(OH)2D levels. Because we measured the same variables on several occasions for each subject, we also used GLM for repeated measures with a within-subject factor (the number of repetitions, specified as study day) and with the between-subject factors—skin type and use of contraceptives [62]. These factors were selected based on their possible association with the investigated VitD measures shown in previous studies. In this model, a conservative measure of significance was used ($p \leq 0.01$) if the assumption of variance homogeneity in all cells was violated. Due to missing VitD measures in the follow-up in some study participants, the procedure GLM for repeated measures was applicable for the 16 women with complete data sets.

Relationships between outcome measures were explored using Spearman correlation.

In most of the calculations all participants were analyzed as an entire group. For examining the UV-induced increase of 25(OH)D and 1,25$(OH)_2$D depending on the VitD status at baseline, two subgroups with 25(OH)D baseline <50 nmol/L (n = 7) and ≥50 nmol/L (n = 13) were compared.

Results

Baseline characteristics

The demographic and psychometric characteristics of the study group at baseline including age, BMI, use of oral contraceptives, smoking status, daily intake of VitD, BDI and POMS score are reported in Table 1.

Table 1. Baseline characteristics of subject group.

	n = 20
age (years)	23.0±1.2 [21–25]
BMI (kg/m^2)	21.2±2.6 [17.5–27.7]
oral contraceptives, % (n)	60% (12)
smokers, % (n)	10% (2)
Fitzpatrick skin types I /II/III (n)	10% (2)/30% (6)/60% (12)
daily dietary VitD intake (μg/d)	3.0±2.2 [0.7–10.0]
baseline BDI total score	4.0±2.1 [0–8]
baseline POMS total score	0.5±14.3 [–15–49]

n, number; s.d., standard deviation; BMI, body mass index; VitD, Vitamin D; BDI, Beck Depression Inventory; POMS, Profile of Mood States; absolute values are given as mean±s.d. [range]

doi:10.1371/journal.pone.0159040.t001

All women scored low on the BDI, with scores ranging from 0–8. The mean POMS total score indicated good well-being und functioning. Fitzpatrick skin types II and III were represented in 90% of the participants.

Table 2 shows the baseline mean total serum levels of 25(OH)D and 1,25(OH)$_2$D. Looking at individual values, seven of the 20 participants had a VitD deficiency, with 25(OH)D levels below 50 nmol/L, and twelve an insufficiency with concentrations between 50 and 75 nmol/L. The daily VitD intake correlated moderately with baseline 1,25(OH)$_2$D concentration (n = 20, rho = 0.458, p = 0.042), but not with the 25(OH)D concentration (n = 20, rho = 0.088, p = 0.712).

To explain this variance in baseline 25(OH)D and 1,25(OH)$_2$D by possible confounds such as the use of oral contraceptives, skin type (as factors) and daily nutritional VitD intake (as covariate), we used the GLM. In this model, only a significant main effect of oral contraceptives (F = 6.714, df = 1, p = 0.018) was observed for 1,25(OH)$_2$D but not for 25(OH)D. Post hoc analysis indicated that women using oral contraceptives (n = 12) had higher average

Table 2. 25-hydroxyvitamin D and 1,25-dihydroxyvitamin D at baseline, day 8, day 36, day 50 and change from baseline.

		baseline (n = 20)	day 8 (n = 20)	day 36 (n = 17)	day 50 (n = 18)
**25(OH)D, nmol/L**	mean±s.d.	54.4±24.1	68.3±18.2	62.0±22.8	60.3±21.6
	Median	54.7	67.0	62.4	63.1
	Range	12.5-122.1	43.7-119.3	24.0-125.0	22.3-116.5
	Δ CfB		13.9±9.5	8.8±8.4	5.2±10.0
	95% CI		9.4 to 18.4	4.5 to 13.1	0.2 to 10.1
	p value*		<0.001	0.001	0.044
**1,25(OH)$_2$D,pmol/L**	mean±s.d.	130.9±35.8	157.1±49.8	129.8±44.9	125.7±33.3
	Median	138.5	167.0	125.0	118.0
	Range	71-201	78-236	38–208	71–182
	Δ CfB		26.2±40.4	-7.1±33.2	-11.0±36.6
	95% CI		7.2 to 45.1	-24.2 to 9.9	-29.2 to 7.2
	p value*		0.009	0.39	0.22

25(OH)D, 25-hydroxy vitamin D; 1,25(OH)$_2$D, 1,25-dihydroxyvitamin D; s.d., standard deviation; CI, confidence interval; CfB, change from baseline
*p values for the paired t-test versus baseline; n, number; Δ, delta

doi:10.1371/journal.pone.0159040.t002

$1,25(OH)_2D$ levels at baseline (145.8±31.6 vs. 108.6±31.1 pmol/L, T = -2.591, p = 0.018), but similar 25(OH)D values (57.4±27.4 and 50.0±18.8 nmol/L).

Interestingly, BDI baseline values correlated moderately and negatively with baseline concentrations of $1,25(OH)_2D$ (n = 20, rho = -0.597, p = 0.005) and of 25(OH)D (n = 20, rho = -0.542, p = 0.013), whereas both VitD metabolites were not correlated (rho = 0.333, not significant).

UVR effects on Vitamin D status

A low cumulative erythemally weighted dose of UV irradiation of 3.5 SED within one week was given to only two participants (skin type I). The remaining 18 women (skin types II and III) were subjected to a cumulative dose of 4.375 SED. Overall, after the three UVR exposures, a significant increase in 25(OH)D and $1,25(OH)_2D$ levels was observed at study day 8 (Table 2). Individual courses of 25(OH)D levels throughout the study are shown in Fig 2. Only the single participant with an ideal level at baseline did not experience an increase after UVR exposures (Fig 2). Whereas the mean serum level of 25(OH)D at day 36 and 50 (31 and 45 days after the final UVR exposure) remained significantly higher versus baseline, the concentration of the biologically active metabolite $1,25(OH)_2D$ decreased significantly from day 8 to day 36. At day 50 it was slightly lower than the baseline value (paired t-test versus baseline, Table 2).

GLM for repeated measures (n = 16 women with complete data sets), controlling for skin type, confirmed the significant effect of UVR exposures on 25(OH)D; in the pairwise comparisons the difference versus baseline was significant at day 8 (p = 0.004) and day 36 (p = 0.020), but not at day 50 (p = 0.957).

Notably, the increment of 25(OH)D concentrations above baseline after the three UVR exposures was moderately negatively correlated with the baseline 25(OH)D levels (rho = -0.493, p = 0.027) (Fig 3). Therefore, as expected, a better response (increase in 25(OH)D) was achieved in participants with the lowest baseline 25(OH)D values. In the deficient subjects (<50 nmol/L), increase in 25(OH)D was twice as high (Δ 21.2±10.3 nmol/L) as in the others (Δ 10.0±6.6 nmol/L). Accordingly, this subgroup also showed an average higher increase in 1,25(OH)2D levels at day 8 (Δ 45.1±44.4 vs. Δ 15.9±35.7 pmol/L).

Psychometric measures

There was no correlation between the BDI and POMS total scores (rho = 0.099) at baseline, but a low and significant correlation between the two scales was found at day 8 (rho = 0.446, p = 0.049).

The mean BDI total score decreased significantly from baseline to study day 8 (Table 3).

There was a significant moderate inverse relationship between BDI total score and $1,25(OH)_2D$ or 25(OH)D levels at baseline (rho = -0.597, p = 0.005 and rho = -0.542, p = 0.013, respectively), but not at day 8 (rho = -0.181 and rho = -0.173, respectively).

Next, we analyzed the data from POMS. The mean POMS total score was similar at baseline and day 8 and indicated good well-being and functioning (Table 3). There were also no significant changes for the subscales of POMS (Table 3). However, POMS total score three days after the final UVR exposure, and the increment of $1,25(OH)_2D$, correlated moderately and significantly at this time point (rho = 0.549, p = 0.012). This relationship was mainly due to the POMS subscale vigour/activity. Vigour/activity subscale scores at day 8 and increment in $1,25(OH)_2D$ were strongly and inversely correlated (rho = -0.739, p<0.001) (Fig 4).

Adverse events

The first two irradiations were tolerated without the occurrence of AEs. After the third and last UVR exposure one participant experienced skin dryness, another skin pain, and three mild redness. These AEs were reported as occurring between 1–2 hours after the last UVR exposure.

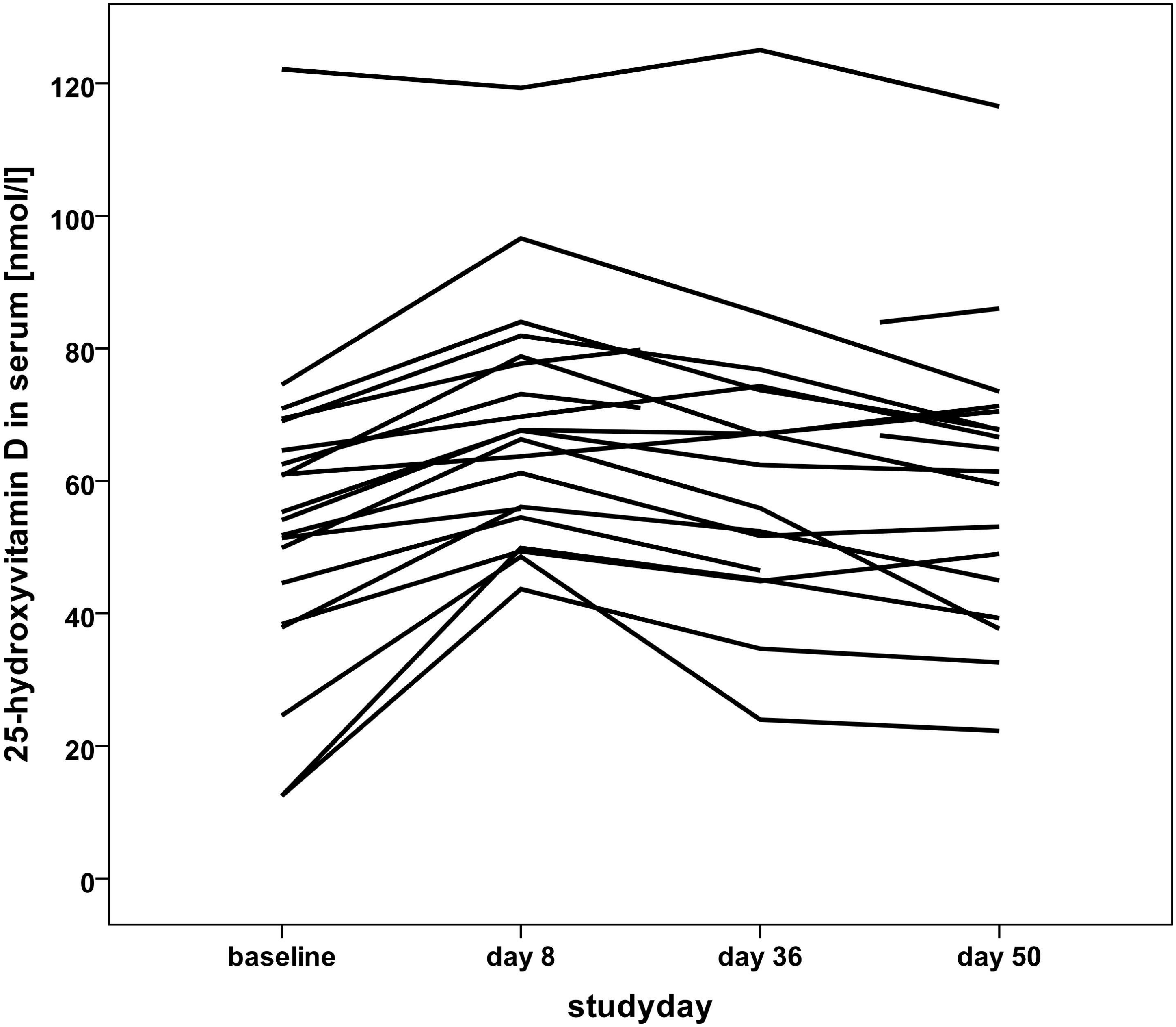

Fig 2. Individual Vitamin D status as expressed by 25-hydroxyvitamin D concentration in plasma during the study.

doi:10.1371/journal.pone.0159040.g002

Two of the three women with diffuse mild redness without defined borders were of skin type II, and one was skin type I. All of the symptoms disappeared within the same evening and no remedial measures were required. Other possible adverse events were not reported. Therefore, the short-term mild skin redness in three women after the last UVR was not classified as an UV-B induced erythema.

Discussion

The first main finding of the MUVY pilot study was that just three serial escalating UVR exposures to whole body area during one week were sufficient in increasing 25(OH)D and 1,25

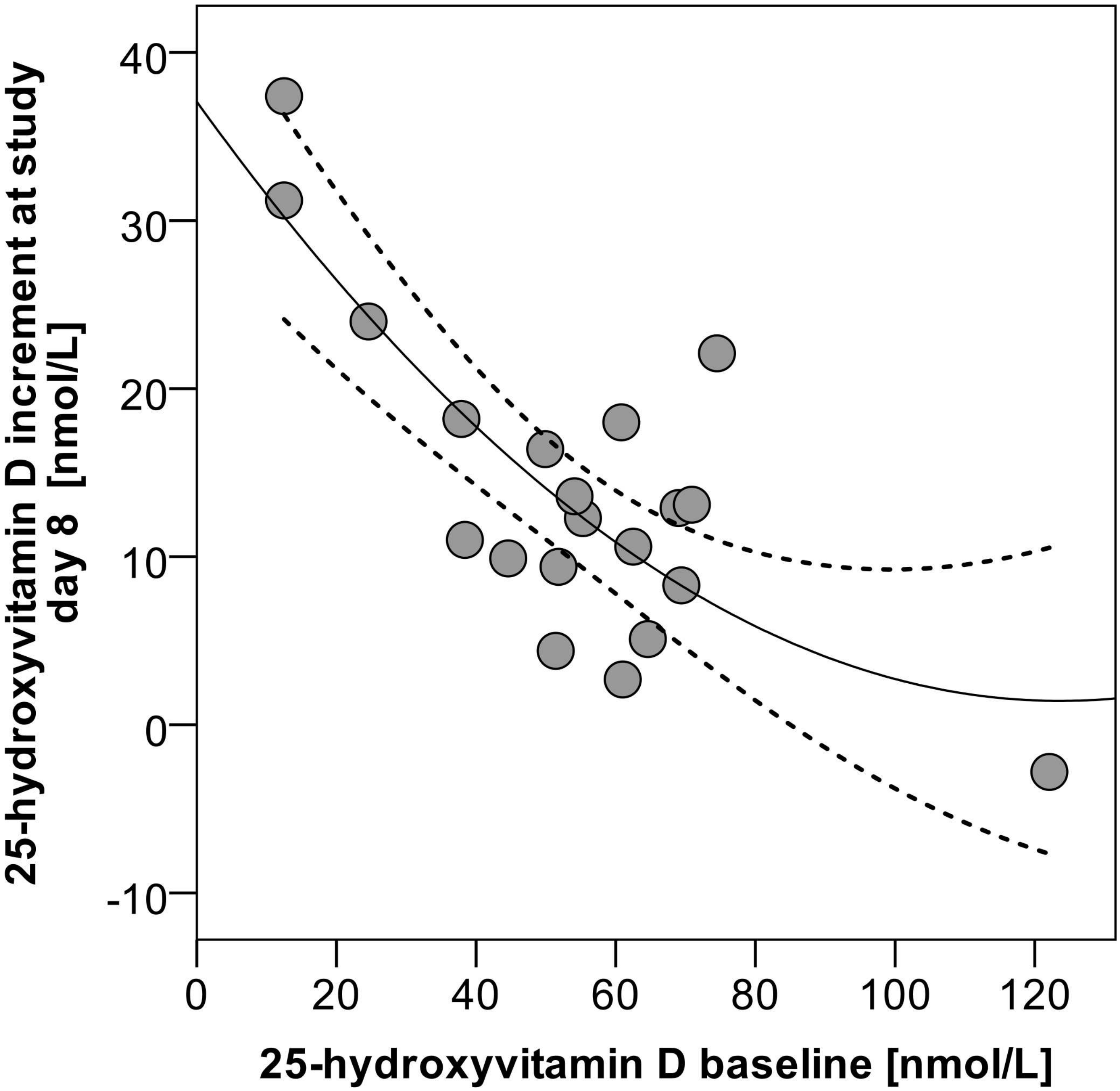

Fig 3. Scatterplot diagram, relationship between increase in 25-hydroxyvitamin D at day 8 and baseline 25-hydroxyvitamin D levels (quadratic regression analysis, $r^2 = 0.607$, p<0.001) The solid line is the quadratic fit, and dashed lines are the 95% confidence limits to the fit.

doi:10.1371/journal.pone.0159040.g003

$(OH)_2D$ total levels in the circulation of young healthy Caucasian women with sun-reactive skin types I–III, and the second was that these effects remained significant for at least six weeks after the last UVR exposure for 25(OH)D (Table 2).

Although we have not pre-examined the individual erythema threshold doses in our study participants for the device used, our schedule to start the whole body UVR exposure with 0,8 SED for skin type I and 1.0 SED for skin types II and III was reliable in avoiding unwanted acute skin reactions such as erythema. Only after the third and last UVR exposure with 1.5

Table 3. BDI and POMS scores at baseline and day 8.

		baseline (n = 20)	day 8 (n = 20)	Wilcoxon test
**BDI score**	mean±s.d.	4.0±2.1	2.6±2.5	Z = -2.386p = 0.017
	range	0–8	0–11	
	median	4.0	3.0	
**POMS total score**	mean±s.d.	0.5±14.3	-0.45±13.2	Z = -0.503p = 0.615
	range	-15-49	-14-34	
	median	-2.0	-5.0	
**POMS depression/anxiety**	mean±s.d.	3.4±5.8	3.6±5.9	Z = -0.143p = 0.886
	Median	1.0	1.0	
	Range	0–26	0–24	
**POMS vigor**	mean±s.d.	10.5±4.7	11.6±4.4	Z = -1.009p = 0.313
	Median	10.0	12.0	
	Range	3–20	3–21	
**POMS fatigue**	mean±s.d.	5.3±3.9	5.0±4.4	Z = -0.415p = 0.678
	Median	5.5	4.5	
	Range	0–16	0–16	
**POMS hostility**	mean±s.d.	2.4±4.6	2.7±2.9	Z = -1.249p = 0.212
	Median	1.0	1.5	
	Range	0–20	0–9	

BDI, Beck Depression Inventory; POMS, Profile of Mood States; s.d., standard deviation; n, number

doi:10.1371/journal.pone.0159040.t003

SED (skin type I) or 1.875 SED (skin types II–III) did five women report adverse events such as skin dryness, skin pain or mild skin redness; all of the symptoms disappeared within the same evening. With respect to the short-time mild skin redness in three women, we would like to assume that this effect cannot be considered as a classical UV-B induced erythema.

Overall, our pilot study showed that for the planning of further research on VitD response to broad band UV-B radiation in young Caucasian women with skin types II–III, an escalating dose schedule for the whole body exposure with a low initial dose and a maximum dose not exceeding 1.875 SED may be considered, practicable and sufficient to induce VitD synthesis in the skin, whilst avoiding unnecessary skin reactions like erythema and tanning. For comparison, in the study by Bogh et al.(2011) four broad band UVB exposures were given in 2 to 3 day intervals to the chest and back (24% body surface area) of 55 selected healthy subjects with baseline 25(OH)D levels ≤50 nmol/L and Fitzpatrick skin types I–IV [30]. The UVB doses tested were between 3.0 and 0.375 SED, with the most pronounced effect on 25(OH)D increase in the group exposed to 3.0 SED. 20% of the study participants in this group developed erythema.

An important issue is determining the frequency and number of UVR exposures necessary during winter to prevent a decline of summer VitD levels and/or to maintain these levels as stable throughout the year. Bogh et al. (2012) explored the effect of a fixed UV-B dose of 1.0 SED applied every second week to 88% body area in 14 healthy subjects (mean age 36 years) during a period of four months, and found that this procedure is sufficient to maintain summer 25 (OH)D concentrations through winter time, whereas the same procedure performed once a week even resulted in significant increases in 25(OH)D levels [31]. On the other hand, Farrar et al. (2011) showed that in 109 Caucasian non-elderly adults from Greater Manchester, UK, with sun-reactive skin types I–IV (individual erythema thresholds between 1.6 SED and 8.2 SED) exposed to a constant UVR dose (1.3 SED) at January/February using a whole body

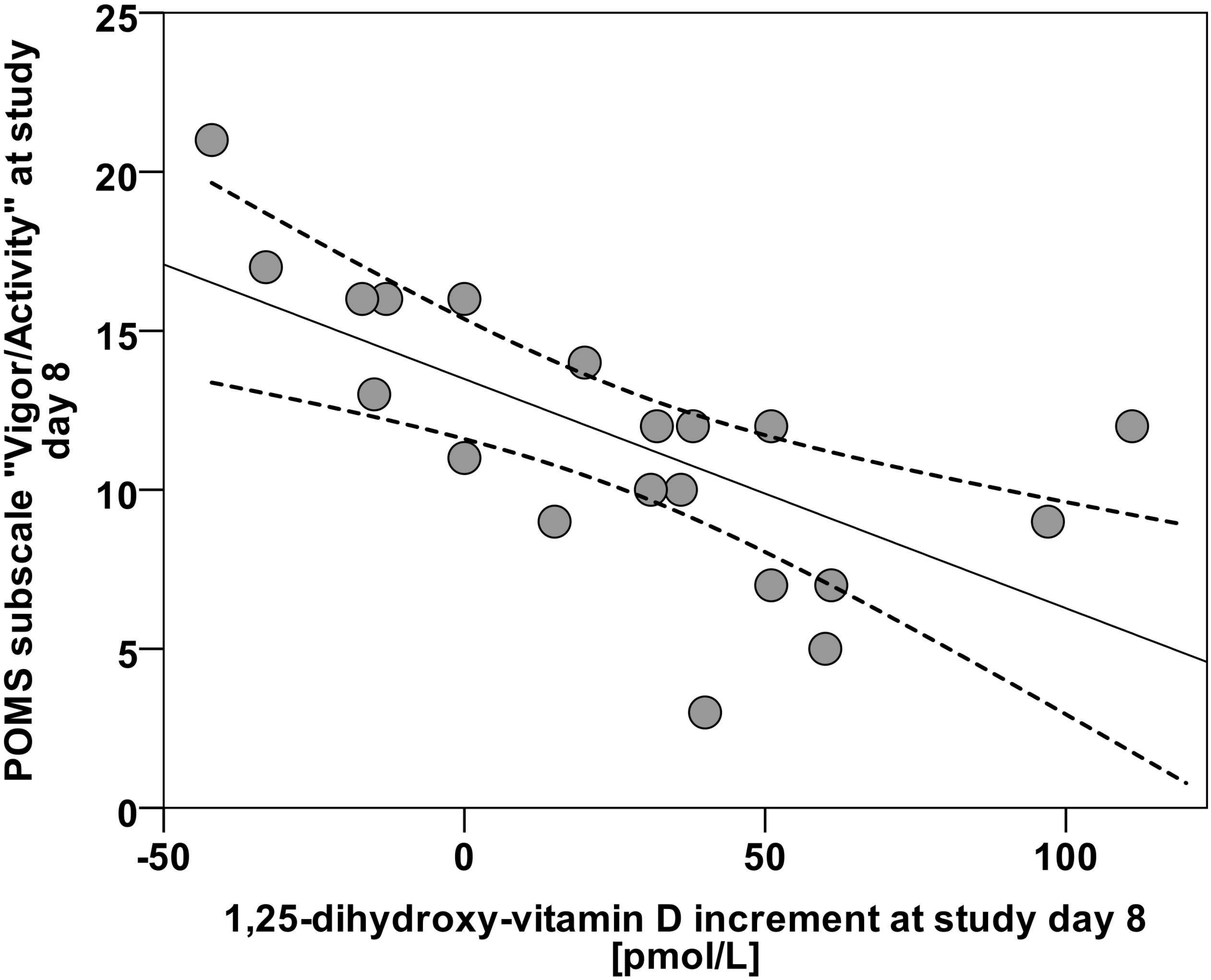

Fig 4. Scatterplot diagram, relationship between POMS subscale Vigor/Activity and the increment in 1,25-dihydroxyvitamin D at day 8 (linear regression analysis, $r^2 = 0.445$, $p<0.001$). The solid line is the linear fit and dashed lines are the 95% confidence limits to the fit.

doi:10.1371/journal.pone.0159040.g004

irradiation cabin (three times weekly for 6 weeks), the weekly gain in mean 25(OH)D levels steadily decreased with only marginal changes in weeks 4, 5 and 6 [36].

In the MUVY pilot study, we found a mean increase in 25(OH)D of 13.9 nmol/L (from baseline to study day 8). This is comparable to effects observed in other UVR exposure studies using fixed doses once per week for two months [23] or four months [31].

Like in the target population of young adults [63], a high rate of VitD de-/insufficiency was detected in the MUVY study participants. Whilst there is debate as to whether skin pigmentation may influence the UVB induced VitD synthesis [64], other studies have shown that the increment of 25(OH)D in serum after UV-B exposures depends upon baseline 25(OH)D levels [65]. In accordance with the data of this literature, we observed a negative correlation between

baseline 25(OH)D levels and its increase after the three UVR exposures (Fig 2). Therefore, as expected, a better response (stronger increase in 25(OH)D) was achieved in participants with the lowest baseline 25(OH)D values. In our subgroup of 7 women with deficient VitD status 25 (OH)D increased from baseline to day 8 by 21.2 nmol/L. Therefore, we can assume that already a small number of whole body suberythemal UVR exposures in winter could improve VitD status, especially in women with VitD insufficiency.

In general, published UVR studies have not conducted follow-up examinations of VitD after the UVR exposures were terminated. However, we are interested in the question of whether the initial rise of 25(OH)D and 1,25$(OH)_2$D in response to UVR exposures can be maintained for a longer time (>4 weeks), or subsequently declines after the treatment has been terminated. Our finding of long-term UVR effects only on 25(OH)D levels in serum (Table 2) may be explained by the fact that its active form 1,25$(OH)_2$D has a short half-life of less than one day compared to three weeks for 25(OH)D [66]. 25(OH)D is fat soluble and storable, and therefore represents a steady VitD state derived from dietary intake and UVR-assisted self-production of the last weeks and months [66]. In this respect, it is notable that after a single $VitD_3$ dose of 100,000 IU, serum levels of 25(OH)D peaked at day 7 and fell slowly to baseline values by day 112 [67].

The possible influence of oral contraception on VitD levels was examined in several studies, mainly observing higher VitD levels in women using oral contraceptives [68–71]. In the MUVY study, women with oral contraceptives showed significantly higher 1,25$(OH)_2$ baseline levels. Genetic polymorphisms in the VitD receptor and increased levels of VitD binding protein seem to play a role [70, 72].

To our knowledge there are no systematic studies investigating the effects of UVR exposures on the VitD status, and different aspects of well-being in patients with clear psychiatric diagnosis of major/minor depression or seasonal affective disorder but without medical comorbidity in parallel. On the other hand, differences in reported findings in studies with VitD supplementation in patients with depression are likely not only to be affected by study population and dosage schemes, but also by choice of diagnostic criteria, screening tools and self-report questionnaires used. In our opinion, the choice of a suitable self-rating test to detect possible mood changes seems to present a problem.

Therefore, we are also interested in the question of whether UVR exposures in winter months may influence the affective state/well-being of healthy women outside of symptoms/ behaviors which are typical for depressive disorders. Recent or past major and minor depression, according to the DMS IV (Diagnostic and Statistical Manual of Mental Disorders) criteria, was an exclusion criterion of the pilot study. Our study participants had a low mean BDI total score at baseline, which decreased further significantly at day 8 (Table 3). We abstain from interpreting this statistically meaningful result as a clinically significant effect of UVR exposure on depression, since our healthy subjects did not suffer from depressive disorders. On the other hand, associations between improved VitD status by $VitD_3$ supplementation and decrease in BDI total scores, as a measure of depressive symptoms, have been assumed in the frequently cited pilot study by Shipowick et al. [18]. In this study, six women with VitD deficiency and high scores on the BDI-II (range 26–40) completed eight weeks of $VitD_3$ supplementation (5,000 IU/day) [18]. As no significant correlations between BDI-II total score and 25(OH)D serum levels at baseline or after the treatment were found, it is questionable whether a causal relationship exists between the improvement of VitD status and the observed reduction in the severity of depressive symptoms. Medical and psychiatric conditions or medication used by the participants were not sufficiently reported in this study [18].

Our sample of women with good well-being und functioning, despite the majority having low 25(OH)D levels, have limited our ability to detect expected positive effects of UVR

exposure on the affective state. The BDI does not seem to be a suitable instrument for measuring these changes if the study participants are of generally good mental health. On the other hand, POMS as an adjective rating scale which has been used in prospective studies of mood changes both in psychiatric out-patients and subjects without psychiatric conditions could be a helpful psychometric instrument [17, 73].

As indicated by the mean POMS total scores at baseline and day 8 (Table 3), our study group as a whole was in a well-balanced affective status. Nevertheless, our finding of significant positive correlation between POMS total score at day 8 and Δ $1,25(OH)_2D$ seems to indicate that acute changes in biologically active metabolite $1,25(OH)_2D$ could impact upon well-being. Moreover, of all POMS subscales, only vigor/activity was strongly and inversely associated with Δ $1,25(OH)_2D$. Thus, as shown in Fig 4, women with a large increase in $1,25(OH)_2D$ were less vigorous. The interpretation of this finding is difficult. Most VitD and UV-B exposure studies only analyze changes in 25(OH)D, and not in $1,25(OH)_2D$ [22–39]. Given the interaction of $1,25(OH)_2D$ with membrane-based signaling pathways in different cell types [74], as well as the existence of numerous additional VitD metabolites with possible biological activity [2–4], a simple causal relationship should not be assumed. Furthermore, it should also be taken into consideration that the production of neuroactive factor classes other than VitD related compounds is also affected by UVR. Aside from immunoregulatory molecules, neuropeptides, neurotrophins, and neurotransmitters, the CRH-POMC-system (corticotropin-releasing hormone—Pro-opiomelanocortin—system) for example is strongly influenced and regulated by UVR [75].

One important limitation of the MUVY pilot study was that the second assessment of mood and well-being was performed three days after the final UVR exposure. This may have limited our ability to detect potential acute effects of UV irradiation on psychometric measures in healthy young women without clear symptoms of depressed mood or impaired functioning. Notably, most study participants reported spontaneously feeling better after the UVR sessions (less fatigued, more relaxed). Another limitation was that Parathyroid hormone was not measured in this pilot study, but this will be assessed in the subsequent trial.

In summary, we suggest that our findings point to three conclusions

1. The pilot study on healthy young women with predominantly Fitzpatrick skin types II and III showed the feasibility of our schedule with three escalating suberythemal UVR exposures to improve the VitD status not only acutely but also for a longer time, as all twenty participants received every predefined UVR without significant adverse skin reactions.

2. The MUVY pilot study expands the literature on 25(OH)D and/or $1,25(OH)_2D$ levels in serum of apparently healthy subjects in response to different UVR exposures using a device fitted with fluorescent tubes which produces an UVR emission spectrum similar to that of sunlight, by showing that three suberythemal UVR exposures within one week during winter with a dose regime of 1.0–1.5–1.875 SED for women with skin types II and III and 0.8–1.2–1.5 SED for women with skin type I was effective in improving VitD status especially in women with deficient 25(OH)D levels, and in maintaining this effect over a period of at least 6 weeks in the absence of further UVR exposures.

3. Our sample of women with good well-being und functioning despite the majority having low 25(OH)D levels have limited our ability to detect clear positive effects of UVR exposure on the affective state. The BDI seems insufficient as an instrument for measuring these changes if the study participants are of generally good health. As indicated from our findings with POMS, the possible relationship between psychometric measures and VitD metabolites is less simple than frequently assumed.

Our pilot study showed good feasibility in terms of most of the procedures and assessments used. In subsequent larger research projects the schedule may be practicable for investigating the influence of skin type on the acute and long-term effects of UVB on VitD status, including 1,25$(OH)_2$D assessments as well as questionnaires for affective state/well-being in the research program. For possible future application of UVR, for example, in patients suffering from rheumatoid arthritis, a limited number of UVR exposures within one week appears more practical than several weeks of exposure, and might ensure greater compliance. The six-week study of Bogh et al. (2012) with three UVR exposures per week reported a high rate of drop-outs [40].

Supporting Information

S1 Chart. SPSS data set.
(SAV)

S2 Chart. TREND statement Checklist.
(DOCX)

S1 File. Original study protocol.
(PDF)

S2 File. Translation of original study protocol.
(DOCX)

S3 File. Case report form.
(PDF)

Acknowledgments

We thank the participants for their contribution to the study. We would also like to extend our gratitude to S.A. Hoffmann and R.R. Piper for their contribution in editing and proofreading the manuscript.

Author Contributions

Conceived and designed the experiments: MGB MH RU VHZ TB JD HP. Performed the experiments: MGB MH VHZ TB JD. Analyzed the data: MGB MH LF PK JD. Contributed reagents/materials/analysis tools: RU VHZ TB FB GRB JD HP. Wrote the paper: MGB LF PK VHZ TB FB GRB JD HP.

References

1. Holick MF. Sunlight and vitamin D for bone health and prevention of autoimmune diseases, cancers, and cardiovascular disease. Am J Clin Nutr. 2004; 80(6 Suppl):1678S–88S. PMID: 15585788.
2. Norman AW, Silva FR. Structure function studies: identification of vitamin D analogs for the ligand-binding domains of important proteins in the vitamin D-endocrine system. Rev Endocr Metab Disord. 2001; 2(2):229–38. PMID: 11705328.
3. Slominski AT, Kim TK, Li W, Postlethwaite A, Tieu EW, Tang EK, et al. Detection of novel CYP11A1-derived secosteroids in the human epidermis and serum and pig adrenal gland. Sci Rep. 2015; 5:14875. doi: 10.1038/srep14875 PMID: 26445902; PubMed Central PMCID: PMCPMC4597207.
4. Slominski AT, Kim TK, Shehabi HZ, Semak I, Tang EK, Nguyen MN, et al. In vivo evidence for a novel pathway of vitamin D_3 metabolism initiated by P450scc and modified by CYP27B1. FASEB J. 2012; 26 (9):3901–15. doi: 10.1096/fj.12-208975 PMID: 22683847; PubMed Central PMCID: PMCPMC3425822.
5. Hilger J, Friedel A, Herr R, Rausch T, Roos F, Wahl DA, et al. A systematic review of vitamin D status in populations worldwide. Br J Nutr. 2014; 111(1):23–45. doi: 10.1017/S0007114513001840 PMID: 23930771.

6. Richter K, Breitner S, Webb AR, Huth C, Thorand B, Kift R, et al. Influence of external, intrinsic and individual behaviour variables on serum 25(OH)D in a German survey. J Photochem Photobiol B. 2014; 140:120–9. doi: 10.1016/j.jphotobiol.2014.07.018 PMID: 25116947.
7. Anglin RE, Samaan Z, Walter SD, McDonald SD. Vitamin D deficiency and depression in adults: systematic review and meta-analysis. Br J Psychiatry. 2013; 202:100–7. doi: 10.1192/bjp.bp.111.106666 PMID: 23377209.
8. Ju SY, Lee YJ, Jeong SN. Serum 25-hydroxyvitamin D levels and the risk of depression: a systematic review and meta-analysis. J Nutr Health Aging. 2013; 17(5):447–55. doi: 10.1007/s12603-012-0418-0 PMID: 23636546.
9. Hoogendijk WJ, Lips P, Dik MG, Deeg DJ, Beekman AT, Penninx BW. Depression is associated with decreased 25-hydroxyvitamin D and increased parathyroid hormone levels in older adults. Arch Gen Psychiatry. 2008; 65(5):508–12. doi: 10.1001/archpsyc.65.5.508 PMID: 18458202.
10. Milaneschi Y, Shardell M, Corsi AM, Vazzana R, Bandinelli S, Guralnik JM, et al. Serum 25-hydroxyvitamin D and depressive symptoms in older women and men. J Clin Endocrinol Metab. 2010; 95 (7):3225–33. doi: 10.1210/jc.2010-0347 PMID: 20444911; PubMed Central PMCID: PMCPMC2928895.
11. Milaneschi Y, Hoogendijk W, Lips P, Heijboer AC, Schoevers R, van Hemert AM, et al. The association between low vitamin D and depressive disorders. Mol Psychiatry. 2013. doi: 10.1038/mp.2013.36 PMID: 23568194.
12. Annweiler C, Rastmanesh R, Richard-Devantoy S, Beauchet O. The role of vitamin D in depression: from a curious idea to a therapeutic option. J Clin Psychiatry. 2013; 74(11):1121–2. doi: 10.4088/JCP.13ac08783 PMID: 24330899.
13. Naveilhan P, Neveu I, Wion D, Brachet P. 1,25-Dihydroxyvitamin D3, an inducer of glial cell line-derived neurotrophic factor. Neuroreport. 1996; 7(13):2171–5. PMID: 8930983.
14. Zhang X, Zhang Z, Xie C, Xi G, Zhou H, Zhang Y, et al. Effect of treatment on serum glial cell line-derived neurotrophic factor in depressed patients. Prog Neuropsychopharmacol Biol Psychiatry. 2008; 32(3):886–90. doi: 10.1016/j.pnpbp.2008.01.004 PMID: 18262703.
15. Puchacz E, Stumpf WE, Stachowiak EK, Stachowiak MK. Vitamin D increases expression of the tyrosine hydroxylase gene in adrenal medullary cells. Brain Res Mol Brain Res. 1996; 36(1):193–6. PMID: 9011759.
16. Humble MB. Vitamin D, light and mental health. J Photochem Photobiol B. 2010; 101(2):142–9. doi: 10.1016/j.jphotobiol.2010.08.003 PMID: 20800506.
17. Harris S, Dawson-Hughes B. Seasonal mood changes in 250 normal women. Psychiatry Res. 1993; 49(1):77–87. PMID: 8140183.
18. Shipowick CD, Moore CB, Corbett C, Bindler R. Vitamin D and depressive symptoms in women during the winter: a pilot study. Appl Nurs Res. 2009; 22(3):221–5. doi: 10.1016/j.apnr.2007.08.001 PMID: 19616172.
19. Bertone-Johnson ER, Powers SI, Spangler L, Larson J, Michael YL, Millen AE, et al. Vitamin D supplementation and depression in the women's health initiative calcium and vitamin D trial. Am J Epidemiol. 2012; 176(1):1–13. doi: 10.1093/aje/kwr482 PMID: 22573431; PubMed Central PMCID: PMCPMC3385159.
20. Mozaffari-Khosravi H, Nabizade L, Yassini-Ardakani SM, Hadinedoushan H, Barzegar K. The effect of 2 different single injections of high dose of vitamin D on improving the depression in depressed patients with vitamin D deficiency: a randomized clinical trial. J Clin Psychopharmacol. 2013; 33(3):378–85. doi: 10.1097/JCP.0b013e31828f619a PMID: 23609390.
21. Holick MF. Vitamin D: The underappreciated D-lightful hormone that is important for skeletal and cellular health. Current Opinion in Endocrinology, Diabetes and Obesity. 2002; 9(1):87–98.
22. Chel VG, Ooms ME, Popp-Snijders C, Pavel S, Schothorst AA, Meulemans CC, et al. Ultraviolet irradiation corrects vitamin D deficiency and suppresses secondary hyperparathyroidism in the elderly. J Bone Miner Res. 1998; 13(8):1238–42. doi: 10.1359/jbmr.1998.13.8.1238 PMID: 9718191.
23. Chel VG, Ooms ME, Pavel S, de Gruijl F, Brand A, Lips P. Prevention and treatment of vitamin D deficiency in Dutch psychogeriatric nursing home residents by weekly half-body UVB exposure after showering: a pilot study. Age Ageing. 2011; 40(2):211–4. doi: 10.1093/ageing/afq159 PMID: 21183468.
24. Corless D, Gupta SP, Switala S, Barragry JM, Boucher BJ, Cohen RD, et al. Response of plasma-25-hydroxyvitamin D to ultraviolet irradiation in long-stay geriatric patients. Lancet. 1978; 2(8091):649–51. PMID: 80577.
25. Chuck A, Todd J, Diffey B. Subliminal ultraviolet-B irradiation for the prevention of vitamin D deficiency in the elderly: a feasibility study. Photodermatol Photoimmunol Photomed. 2001; 17(4):168–71. PMID: 11499538.

26. Snell AP, MacLennan WJ, Hamilton JC. Ultra-violet irradiation and 25-hydroxy-vitamin D levels in sick old people. Age Ageing. 1978; 7(4):225–8. PMID: 727072.

27. Chandra P, Wolfenden LL, Ziegler TR, Tian J, Luo M, Stecenko AA, et al. Treatment of vitamin D deficiency with UV light in patients with malabsorption syndromes: a case series. Photodermatol Photoimmunol Photomed. 2007; 23(5):179–85. doi: 10.1111/j.1600-0781.2007.00302.x PMID: 17803596; PubMed Central PMCID: PMCPMC2846322.

28. Wortsman J, Matsuoka LY, Chen TC, Lu Z, Holick MF. Decreased bioavailability of vitamin D in obesity. Am J Clin Nutr. 2000; 72(3):690–3. PMID: 10966885.

29. Armas LA, Dowell S, Akhter M, Duthuluru S, Huerter C, Hollis BW, et al. Ultraviolet-B radiation increases serum 25-hydroxyvitamin D levels: the effect of UVB dose and skin color. J Am Acad Dermatol. 2007; 57(4):588–93. doi: 10.1016/j.jaad.2007.03.004 PMID: 17637484.

30. Bogh MK, Schmedes AV, Philipsen PA, Thieden E, Wulf HC. Vitamin D production depends on ultraviolet-B dose but not on dose rate: a randomized controlled trial. Exp Dermatol. 2011; 20(1):14–8. doi: 10.1111/j.1600-0625.2010.01201.x PMID: 21158934.

31. Bogh MK, Schmedes AV, Philipsen PA, Thieden E, Wulf HC. A small suberythemal ultraviolet B dose every second week is sufficient to maintain summer vitamin D levels: a randomized controlled trial. Br J Dermatol. 2012; 166(2):430–3. doi: 10.1111/j.1365-2133.2011.10697.x PMID: 22013924.

32. Edström DW, Linder J, Wennersten G, Brismar K, Ros AM. Phototherapy with ultraviolet radiation: a study of hormone parameters and psychological effects. J Eur Acad Dermatol Venereol. 2010; 24 (4):403–9. doi: 10.1111/j.1468-3083.2009.03423.x PMID: 19778361.

33. Harari M, Dramsdahl E, Shany S, Baumfeld Y, Ingber A, Novack V, et al. Increased vitamin D serum levels correlate with clinical improvement of rheumatic diseases after Dead Sea climatotherapy. Isr Med Assoc J. 2011; 13(4):212–5. PMID: 21598808.

34. Gloth FM, Alam W, Hollis B. Vitamin D vs broad spectrum phototherapy in the treatment of seasonal affective disorder. J Nutr Health Aging. 1999; 3(1):5–7. PMID: 10888476.

35. Libon F, Cavalier E, Nikkels AF. Skin color is relevant to vitamin D synthesis. Dermatology. 2013; 227 (3):250–4. doi: 10.1159/000354750 PMID: 24134867.

36. Farrar MD, Kift R, Felton SJ, Berry JL, Durkin MT, Allan D, et al. Recommended summer sunlight exposure amounts fail to produce sufficient vitamin D status in UK adults of South Asian origin. Am J Clin Nutr. 2011; 94(5):1219–24. doi: 10.3945/ajcn.111.019976 PMID: 21918215.

37. Farrar MD, Webb AR, Kift R, Durkin MT, Allan D, Herbert A, et al. Efficacy of a dose range of simulated sunlight exposures in raising vitamin D status in South Asian adults: implications for targeted guidance on sun exposure. Am J Clin Nutr. 2013; 97(6):1210–6. doi: 10.3945/ajcn.112.052639 PMID: 23615828.

38. Osmancevic A, Gillstedt M, Landin-Wilhelmsen K, Wennberg Larkö AM, Larkö O, Holick MF, et al. Size of the exposed body surface area, skin erythema and body mass index predict skin production of vitamin D. J Photochem Photobiol B. 2015; 149:224–9. doi: 10.1016/j.jphotobiol.2015.06.008 PMID: 26093795.

39. Juzeniene A, Baturaite Z, Lagunova Z, Grigalavicius M, Porojnicu AC, Bruland Ø, et al. Influence of multiple UV exposures on serum cobalamin and vitamin D levels in healthy females. Scand J Public Health. 2015. doi: 10.1177/1403494815572206 PMID: 25740614.

40. Bogh MK, Gullstrand J, Svensson A, Ljunggren B, Dorkhan M. Narrowband ultraviolet B three times per week is more effective in treating vitamin D deficiency than 1600 IU oral vitamin D_3 per day: a randomized clinical trial. Br J Dermatol. 2012; 167(3):625–30. doi: 10.1111/j.1365-2133.2012.11069.x PMID: 22632734.

41. Holick MF. Vitamin D deficiency. N Engl J Med. 2007; 357(3):266–81. doi: 10.1056/NEJMra070553 PMID: 17634462.

42. Lips P. Relative value of 25(OH)D and 1,25(OH)2D measurements. J Bone Miner Res. 2007; 22 (11):1668–71. doi: 10.1359/jbmr.070716 PMID: 17645404.

43. Bouillon RA, Auwerx JH, Lissens WD, Pelemans WK. Vitamin D status in the elderly: seasonal substrate deficiency causes 1,25-dihydroxycholecalciferol deficiency. Am J Clin Nutr. 1987; 45(4):755–63. PMID: 3494392.

44. Lancaster GA, Dodd S, Williamson PR. Design and analysis of pilot studies: recommendations for good practice. J Eval Clin Pract. 2004; 10(2):307–12. doi: 10.1111/j.2002.384.doc.x PMID: 15189396.

45. Johanson GA, Brooks GP. Initial Scale Development: Sample Size for Pilot Studies. Educational and Psychological Measurements. 2010; 70(3):394–400.

46. Webb AR, Kline L, Holick MF. Influence of season and latitude on the cutaneous synthesis of vitamin D3: exposure to winter sunlight in Boston and Edmonton will not promote vitamin D3 synthesis in human skin. J Clin Endocrinol Metab. 1988; 67(2):373–8. PMID: 2839537.

47. Taylor DK, Anstey AV, Coleman AJ, Diffey BL, Farr PM, Ferguson J, et al. Guidelines for dosimetry and calibration in ultraviolet radiation therapy: a report of a British Photodermatology Group workshop. Br J Dermatol. 2002; 146(5):755–63. PMID: 12000370.
48. CIE. Erythema reference action spectrum and standard erythema dose. Commission Internationale De L'Eclairage, Technical Report, CIE S 007/E-1998.
49. Bundesministerium für Umwelt, Verordnung zum Schutz vor schädlichen Wirkungen künstlicher ultravioletter Strahlung (UV-Schutz-Verordnung, UVSV), Bundesregierung Deutschland 2011.
50. Fitzpatrick TB. The validity and practicality of sun-reactive skin types I through VI. Arch Dermatol. 1988; 124(6):869–71. PMID: 3377516.
51. Sachdeva S. Fitzpatrick skin typing: applications in dermatology. Indian J Dermatol Venereol Leprol. 2009; 75(1):93–6. PMID: 19172048.
52. DIfE. German Food Propensity Questionnaire (GFPQ), Deutsches Instituts für Ernährungsforschung Potsdam-Rehbrücke (DIfE) 2008–2013, Available: https://efbo.dife.de/gfpq/page/de, accessed 16-Sep-2013.
53. age and gender adapted portion sizes: "Die aid Ernährungspyramide—Richtig essen lehren und lernen", information service on nutrition, agriculture and consumer protection, Germany, Available: http://www.aid.de/downloads/pyramide_tabelle_portionsgroesse.pdf, accessed 16-Sep-2013.
54. Nutritional content online calculator, Available: http://www.naehrwertrechner.de/, accessed 16-Sep-2013.
55. Beck AT, Ward CH, Mendelson M, Mock J, Erbaugh J. An inventory for measuring depression. Arch Gen Psychiatry. 1961; 4:561–71. PMID: 13688369.
56. Seggar LB, Lambert MJ, Hansen NB. Assessing Clinical Significance: Application to the Beck Depression Inventory. Behavior Therapy. 2002; 33(2):253–69.
57. McNair D, Lorr M, Droppleman L. POMS Manual. San Diego: Educational and Industrial Testing Service; 1971.
58. Albani C, Blaser G, Geyer M, Schmutzer G, Hinz A, Bailer H, et al. [Validation and standardization of the "Questionnaire for Assessing Subjective Physical Well-Being" by Kolip and Schmidt in a representative German sample]. Psychother Psychosom Med Psychol. 2006; 56(3–4):172–81. PMID: 16802423.
59. Dawson-Hughes B, Heaney RP, Holick MF, Lips P, Meunier PJ, Vieth R. Estimates of optimal vitamin D status. Osteoporos Int. 2005; 16(7):713–6. doi: 10.1007/s00198-005-1867-7 PMID: 15776217.
60. Labor Berlin—Kompetenz von Charité und Vivantes. 1 -O-DR, Available: http://www.laborberlin.com/leistungsverzeichnis.html?k=8&ue1=177&u=956&index=, accessed 21-Feb-2016.
61. National Institutes of Health and National Cancer Institute, Common Terminology Criteria for Adverse Events (CTCAE), U.S. Department of Health and Human Services 2010.
62. Ho R. Handbook of Univariate and Multivariate Data Analysis and Interpretation with SPSS, Chapman & Hall/CRC is an imprint of Taylor & Francis Group, USA, 2006, pp 117–182.
63. Linseisen J, Bechthold A, Bischoff-Ferrari HA, al. e. Vitamin D und Prävention ausgewählter chronischer Krankheiten–Stellungnahme. In: V. DGfEe, editor. Bonn2011.
64. Xiang F, Lucas R, de Gruijl F, Norval M. A systematic review of the influence of skin pigmentation on changes in the concentrations of vitamin D and 25-hydroxyvitamin D in plasma/serum following experimental UV irradiation. Photochem Photobiol Sci. 2015; 14(12):2138–46. doi: 10.1039/c5pp00168d PMID: 26548800.
65. Bogh MK, Schmedes AV, Philipsen PA, Thieden E, Wulf HC. Vitamin D production after UVB exposure depends on baseline vitamin D and total cholesterol but not on skin pigmentation. J Invest Dermatol. 2010; 130(2):546–53. doi: 10.1038/jid.2009.323 PMID: 19812604.
66. Holick MF. The use and interpretation of assays for vitamin D and its metabolites. J Nutr. 1990; 120 Suppl 11:1464–9. PMID: 2243289.
67. Heaney RP, Armas LA, Shary JR, Bell NH, Binkley N, Hollis BW. 25-Hydroxylation of vitamin D3: relation to circulating vitamin D3 under various input conditions. Am J Clin Nutr. 2008; 87(6):1738–42. PMID: 18541563.
68. Hronek M, Poljaková G. [Effect of hormonal contraception on levels of vitamins in the body]. Ceska Gynekol. 2004; 69(5):397–401. PMID: 15587897.
69. Harris SS, Dawson-Hughes B. The association of oral contraceptive use with plasma 25-hydroxyvitamin D levels. J Am Coll Nutr. 1998; 17(3):282–4. PMID: 9627916.
70. Møller UK, Streym S, Jensen LT, Mosekilde L, Schoenmakers I, Nigdikar S, et al. Increased plasma concentrations of vitamin D metabolites and vitamin D binding protein in women using hormonal

contraceptives: a cross-sectional study. Nutrients. 2013; 5(9):3470–80. doi: 10.3390/nu5093470 PMID: 24013463; PubMed Central PMCID: PMCPMC3798915.

71. van Hoof HJ, de Sévaux RG, van Baelen H, Swinkels LM, Klipping C, Ross HA, et al. Relationship between free and total 1,25-dihydroxyvitamin D in conditions of modified binding. Eur J Endocrinol. 2001; 144(4):391–6. PMID: 11275949.

72. Pinter B, Kocijancic A, Marc J, Andolsek-Jeras L, Prezelj J. Vitamin D receptor gene polymorphism and bone metabolism during low-dose oral contraceptive use in young women. Contraception. 2003; 67(1):33–7. PMID: 12521655.

73. Kasper S, Rogers SL, Yancey A, Schulz PM, Skwerer RG, Rosenthal NE. Phototherapy in individuals with and without subsyndromal seasonal affective disorder. Arch Gen Psychiatry. 1989; 46(9):837–44. PMID: 2774849.

74. Norman AW, Bouillon R. Vitamin D nutritional policy needs a vision for the future. Exp Biol Med (Maywood). 2010; 235(9):1034–45. doi: 10.1258/ebm.2010.010014 PMID: 20667908.

75. Slominski A, Wortsman J. Neuroendocrinology of the skin. Endocr Rev. 2000; 21(5):457–87. doi: 10.1210/edrv.21.5.0410 PMID: 11041445.
